# Does It Pay Off to Explicitly Link Functional Gene Expression to Denitrification Rates in Reaction Models?

**DOI:** 10.3389/fmicb.2021.684146

**Published:** 2021-06-18

**Authors:** Anna Störiko, Holger Pagel, Adrian Mellage, Olaf A. Cirpka

**Affiliations:** ^1^Center for Applied Geoscience, University of Tübingen, Tübingen, Germany; ^2^Biogeophysics, Institute of Soil Science and Land Evaluation, University of Hohenheim, Stuttgart, Germany

**Keywords:** denitrification, biogeochemical modeling, functional genes, transcripts, enzyme-based modeling, bayesian inference

## Abstract

Environmental omics and molecular-biological data have been proposed to yield improved quantitative predictions of biogeochemical processes. The abundances of functional genes and transcripts relate to the number of cells and activity of microorganisms. However, whether molecular-biological data can be quantitatively linked to reaction rates remains an open question. We present an enzyme-based denitrification model that simulates concentrations of transcription factors, functional-gene transcripts, enzymes, and solutes. We calibrated the model using experimental data from a well-controlled batch experiment with the denitrifier *Paracoccous denitrificans*. The model accurately predicts denitrification rates and measured transcript dynamics. The relationship between simulated transcript concentrations and reaction rates exhibits strong non-linearity and hysteresis related to the faster dynamics of gene transcription and substrate consumption, relative to enzyme production and decay. Hence, assuming a unique relationship between transcript-to-gene ratios and reaction rates, as frequently suggested, may be an erroneous simplification. Comparing model results of our enzyme-based model to those of a classical Monod-type model reveals that both formulations perform equally well with respect to nitrogen species, indicating only a low benefit of integrating molecular-biological data for estimating denitrification rates. Nonetheless, the enzyme-based model is a valuable tool to improve our mechanistic understanding of the relationship between biomolecular quantities and reaction rates. Furthermore, our results highlight that both enzyme kinetics (i.e., substrate limitation and inhibition) and gene expression or enzyme dynamics are important controls on denitrification rates.

## 1. Introduction

Modern high-throughput molecular-biological techniques and omics methods provide insights into the abundance, activity, and metabolic function of microorganisms in environmental systems (Bouchez et al., 2016; Starke et al., 2019). Because microbial transformations play a key role in biogeochemical cycling (Kuypers et al., 2018) and contaminant degradation (Dong et al., 2019), reactive-transport models require an adequate quantification of microbial dynamics. Numerous laboratory and field studies (Bælum et al., 2008; Bowen et al., 2014; Anantharaman et al., 2016; Wegner et al., 2019) indicate that quantifying the abundance of genes and gene transcripts has the potential to improve the description of microbially mediated reactions implemented in reactive-transport models.

Transcript-to-gene ratios and relative gene expression levels have been suggested as a direct measure of microbial activity (Nicolaisen et al., 2008; Freitag and Prosser, 2009; Brow et al., 2013; Monard et al., 2013; Rohe et al., 2020). However, assuming a unique (e.g., linear) relationship neglects important factors that modulate reaction rates such as post-transcriptional and post-translational regulation, different time-scales of transcript and enzyme dynamics, and substrate limitation (Moran et al., 2013). Therefore, integrating a mechanistic description of the aforementioned processes into models could help to better establish a mechanistic link between transcript concentrations and reaction rates. Most reactive-transport models do not explicitly account for genes or transcription and thus cannot integrate molecular-biological datasets into quantitative validation frameworks. Addressing this challenge and integrating omics data into reactive-transport modeling has been proposed as a way to improve our understanding of the dynamic behavior of biogeochemical systems (Li et al., 2017a).

In a first approach to integrate genetic information, genome-scale metabolic models have been coupled to reactive transport, successfully predicting reaction rates under variable environmental conditions without the need of calibration (Scheibe et al., 2009). Though computationally intensive, genome-scale metabolic networks provide a powerful approach for cases in which reactions are dominated by a single, well-known organism or a small group of organisms, as in the case of uranium reduction. Other studies have focused on a single metabolic pathway of an organism, simulating the regulatory chain, including transcription factors, mRNA, enzymes, substrate consumption, and growth kinetics (Koutinas et al., 2011; Bælum et al., 2013).

Natural biogeochemical cycles, such as the nitrogen cycle, are mediated by a diverse group of organisms, of which only a small percentage can be cultivated. Under these circumstances, genome-scale models and models focusing on the functionality of a single microbial strain become impractical. Instead, approaches that target metabolic pathways rather than processes at the scale of a single cell may capture the explicit contribution of the non-cultivatable majority within a natural microbial community. Thus, the alternative *gene-centric* approach focuses on the functionality represented by certain genes instead of specific organisms. By using functional-gene abundances as a proxy for biomass with a certain function, gene-centric approaches can easily be integrated into existing biogeochemical and reactive-transport models (Reed et al., 2014, 2015). Genomic data provide information on metabolic potential, but not on actual microbial activity. To account for activity, the gene-centric approach has been further developed to predict concentrations of functional transcripts and enzymes (Louca et al., 2016; Li et al., 2017c; Song et al., 2017). In one of the studies, the model was validated using metatranscriptomic and metaproteomic data (Louca et al., 2016). However, because the approach of these authors computes transcript and enzyme concentrations by postprocessing of reactions rates, it only provides a partially mechanistic link between rates, transcription, and enzyme production. Hence, this approach lacks the explicit integration of transcript and enzyme regulatory feedbacks into the biogeochemical model.

The functional-enzyme-based approach, by contrast, simulates concentrations of specific enzymes based on energetic (Li et al., 2017c) or cybernetic (Song and Liu, 2015) considerations, and enzyme concentrations directly regulate reaction rates. None of these modeling approaches represent the actual mechanisms of transcriptional regulation with transcription factors, having the advantage that they do not require special knowledge about the regulatory system. However, the validation of the enzyme-based modeling approach relies on quantitative enzyme data that remain challenging to obtain, particularly for environmental samples. Conversely, the quantification of mRNA via reverse transcription quantitative polymerase chain reaction (RT-qPCR) is well established for both laboratory and field setups.

While reactive-transport models that integrate molecular-biological data are more mechanistic, they unavoidably are more complex than traditional Monod-type formulations. Whether the added complexity actually improves model predictability remains understudied.

In this study, we integrate transcript data into a denitrification reaction model by linking the expression of functional genes to process rates. We explicitly account for transcriptional regulation of denitrification via transcription factors, translating the current conceptual understanding of the regulatory system in *Paracoccus denitrificans* into a quantitative model. The main pathways of nitrogen transformations—denitrification, nitrification, N-fixation, annamox, and dissimilatory nitrate reduction to ammonium (DNRA)—have been extensively studied due to the relevance of reactive nitrogen compounds for ecosystem functioning (Steffen et al., 2015), groundwater contamination (Gutiérrez et al., 2018), eutrophication (Howarth, 2008), and greenhouse gas emissions (Liu and Greaver, 2009). The nitrogen cycle is thus an ideal test case for developing and testing new, enzyme-based models informed by measurements of functional genes and transcripts. Previous gene-centric and enzyme-based modeling studies focused on systems with slow dynamics (kinetics) (Li et al., 2017c; Song et al., 2017; Chavez Rodriguez et al., 2020) or at steady state (Reed et al., 2014; Louca et al., 2016). Here, we apply our model to a dynamic reactive system, that is, one with shifts in the predominant electron accepting species, and inform it with a highly temporally resolved dataset of transcript abundances. Previous studies have highlighted a potentially hysteretic relationship between transcript concentrations and reaction rates (Bælum et al., 2008; Chavez Rodriguez et al., 2020). Our model allows us to further explore the relationship between transcripts, enzymes, and reaction rates and develop mechanistic interpretations of the observations. Via a comparison with a classical Monod-type model formulation that does not integrate transcriptional regulation, we shed light on the potential added benefits of integrating transcript data into (denitrification) reaction models.

## 2. Methods

### 2.1. Conceptual Model Description

We set up a model to simulate the experiments of Qu et al. (2015) performed in well-mixed batch reactors. Qu et al. (2015) monitored aerobic respiration and denitrification coupled to succinate oxidation by the denitrifying organism *P. denitrificans*. Briefly, a series of batch reactors, inoculated with *P. denitrificans*, prepared in an aerobic medium, were amended with 5 mM succinate and 2 mM of ${\text{NO}}_{3}^{-}$. Automated headspace gas measurements (nitrogen gases, O_2_) as well as aqueous-phase measurements of nitrite and optical density (OD) facilitated monitoring time series of concentrations driven by reaction and growth kinetics. In addition, and partly driven by headspace gas concentrations, cells were periodically harvested to measure the concentrations of the functional genes *narG, nirS, norB*, and *nosZ*. Following mRNA extraction, transcript numbers were determined via RT-qPCR using the standard curve method. For further details regarding the experimental procedures, we refer to the original publication (Qu et al., 2015).

**Figure 1:**
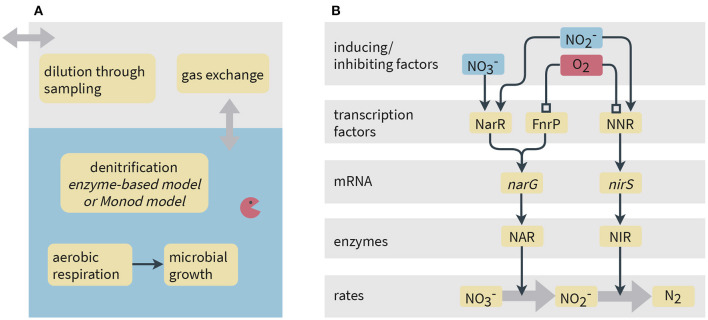
Schematic representation of processes considered in the models. **(A)** Model system and processes implemented common to both the Monod-type and enzyme-based models. **(B)** Simplified representation of gene expression and its link to reaction rates in the enzyme-based model. Narrow arrows represent inducing effects, whereas lines ending in a square denote inhibition. In the model, ${\text{NO}}_{2}^{-}$ instead of NO is used as activating compound for NNR since we do not simulate NO concentrations.

In the experiment, the contribution of the intermediates NO and N_2_O to the mass balance was always less than 1‰. From this experimental observation, we conclude that nitrite reduction was the rate-limiting step. Therefore, we set up a simplified model of denitrification simulating the specific experiments by assuming a two-step process (Figure 1). Therein, the reduction of nitrite via NO and N_2_O to N_2_ was treated as a single reaction step. Denitrification was coupled to the oxidation of succinate as the sole carbon source and electron donor:

(1) $$\begin{array}{l} 7{\text{NO}}_{3}^{-}+{\text{C}}_{4}{\text{H}}_{6}{\text{O}}_{4}\overset{narG}{\to }7{\text{NO}}_{2}^{-}+4{\text{CO}}_{2}+3{\text{H}}_{2}\text{O}, \end{array}$$

(2) $$\begin{array}{l} 14{\text{NO}}_{2}^{-}+14{\text{H}}^{+}+3{\text{C}}_{4}{\text{H}}_{6}{\text{O}}_{4}\overset{nirS}{\to }7{\text{N}}_{2}+12{\text{CO}}_{2}+16{\text{H}}_{2}\text{O}. \end{array}$$

In the reaction equations above, the names of the functional genes linked to the reduction of the nitrogen compounds by *P. denitrificans* are given above the arrows. In the presence of oxygen, aerobic respiration is energetically favored over denitrification:

(3) $$\begin{array}{l} 2{\text{C}}_{4}{\text{H}}_{6}\text{O}4+7{\text{O}}_{2}\to 8{\text{CO}}_{2}+6{\text{H}}_{2}\text{O}. \end{array}$$

As shown in Figure 1, the presence of oxygen is assumed to inhibit denitrification. The electron donor, succinate, was assumed to be primarily assimilated for energy conservation by *P. denitrificans*, and thus we do not consider the incorporation of carbon into biomass during growth. Furthermore, the model considers mass transfer between the liquid and the gas phases via a linear driving force approximation, and the dilution of the gas-phase concentrations by sampling (see section 5 in the Supplementary Material).

We set up two models for comparison: (1) an enzyme-based and (2) a standard Monod-type model. The enzyme-based model considers transcripts and enzymes involved in denitrification reactions as state variables. Reaction rates are directly proportional to enzyme concentrations. The Monod-type model is much simpler and describes denitrification rates using the Monod equation. Therein, the regulation mechanisms and dynamics of catalyzing enzymes are not explicitly reflected, instead the reaction rates are assumed to be directly proportional to the biomass of the denitrifying bacteria.

### 2.2. Governing Equations

Our model represents the transcriptional regulation of denitrification genes by simulating transcription factor concentrations in response to oxygen and nitrogen substrates and their effect on transcript concentrations. For comparison, we also set up a simplified model that omits the explicit representation of transcription factors so that transcription directly depends on the concentrations of signaling molecules (see section 6 in the Supplementary Material). This simplified model can also be interpreted mechanistically (as discussed in section 6.3 of the Supplementary Material), but the assumptions regarding transcriptional regulation differ slightly from the ones presented in the following section.

Several transcription factors regulate the transcription of denitrification genes in *P. denitrificans*, sensing oxygen and nitrogen oxides. The *narG* gene is activated by FnrP and NarR, whereas transcription of *nirS* requires NNR (Gaimster et al., 2018). The FnrP protein directly reacts with oxygen that leads to its inactivation (Crack et al., 2016). NarR responds to nitrate and nitrite, although the underlying mechanism is currently unknown and the sensing might be indirect (Wood et al., 2001; Spiro, 2017). NNR is activated by NO and deactivated by O_2_ (Lee et al., 2006; Gaimster et al., 2018).

We assume that the sum of active and inactive transcription factors remains constant throughout the experiment and simulate all active transcription factors relative to the total concentration. Genes that encode the transcriptional regulators (*fnrP, narR*, and *nnrR*) have been shown to be expressed at similar levels under aerobic and anaerobic conditions in *P. denitrificans* (Giannopoulos et al., 2017), supporting our assumption. We model the fraction of active NarR, *X*_NarR_ [–], as

(4) $$\begin{array}{l} \frac{\text{d}X_{\text{NarR}}}{\text{d}t}=\left(a_{\text{NarR}}^{{\text{NO}}_{3}^{-}}C_{{\text{NO}}_{3}^{-}}+a_{\text{NarR}}^{{\text{NO}}_{2}^{-}}C_{{\text{NO}}_{2}^{-}}\right)\left(1-X_{\text{NarR}}\right)-k_{\text{dec}}^{\text{NarR}}\\ X_{\text{NarR}}, \end{array}$$

where $a_{\text{NarR}}^{{\text{NO}}_{3}^{-}}$ and $a_{\text{NarR}}^{{\text{NO}}_{2}^{-}}$ [M^−1^ s^−1^] are the rate coefficients for activation of NarR by nitrate and nitrite, respectively, and $k_{\text{dec}}^{\text{NarR}}$ [s^−1^] is the dissociation constant of active NarR. The term (1 − *X*_NarR_) represents the inactive fraction of the transcription factor. Binding of oxygen to the transcription factors is described at equilibrium using a Hill function (Gesztelyi et al., 2012). The active fraction of FnrP is

(5) $$\begin{array}{l} X_{\text{FnrP}}=\frac{{I_{\text{FnrP}}}^{p}}{{I_{\text{FnrP}}}^{p}+{C_{{\text{O}}_{2}}}^{p}}, \end{array}$$

with inhibition constant *I*_FnrP_ [M] and Hill coefficient *p* [–]. Experiments have shown that NNR quickly reacts with oxygen, but that its reaction to NO is slower (Lee et al., 2006). We therefore model the inactivation process at equilibrium while accounting for temporal evolution in the activation process. Although NNR actually senses NO, we simulate its activation by nitrite as we do not explicitly model NO concentrations in our two-step representation of denitrification (see conceptual model description above and Figure 1). The activation of NNR is described as follows:

(6) $$\begin{array}{l} \frac{\text{d}{\hat{X}}_{\text{NNR}}}{\text{d}t}=a_{\text{NNR}}C_{{\text{NO}}_{2}^{-}}\left(1-{\hat{X}}_{\text{NNR}}\right)-k_{\text{dec}}^{\text{NNR}}{\hat{X}}_{\text{NNR}}, \end{array}$$

where ${\hat{X}}_{\text{NNR}}$ [–] is the fraction of NNR activated by nitrite (without accounting for inactivation by oxygen), *a*_NNR_ [M^−1^ s^−1^] is the activation rate constant and $k_{\text{dec}}^{\text{NNR}}$ [s^−1^] is the dissociation constant of activated NNR. Taking oxygen inhibition into account, the active fraction of NNR *X*_NNR_ [–] is given by

(7) $$\begin{array}{l} X_{\text{NNR}}=\frac{{I_{\text{NNR}}}^{q}}{{I_{\text{NNR}}}^{q}+{C_{{\text{O}}_{2}}}^{q}}{\hat{X}}_{\text{NNR}}, \end{array}$$

with oxygen inhibition constant *I*_NNR_ [M] and Hill coefficient *q*.

We assume that the transcription rate for gene *i* scales with the fraction of operator sites where all activating transcription factors are bound $f_{\text{act}}^{i}$ (Ingalls, 2013):

(8) $$\begin{array}{l} r_{\text{transcription}}^{i}=\alpha_{i}f_{\text{act}}^{i}B, \end{array}$$

where α_*i*_ [transcripts cell^−1^ s^−1^] is the maximum transcription rate for gene *i* and *B* is the cell density [cells L^−1^]. For *narG* and *nirS*, the fractions of active operator sites are given by equations 9 and 10, respectively:

(9) $$\begin{array}{l} f_{\text{act}}^{nar}=\frac{\frac{X_{\text{FnrP}}X_{\text{NarR}}}{K_{\text{FnrP}}K_{\text{NarR}}}}{1+\frac{X_{\text{FnrP}}}{K_{\text{FnrP}}}+\frac{X_{\text{NarR}}}{K_{\text{NarR}}}+\frac{X_{\text{FnrP}}X_{\text{NarR}}}{K_{\text{FnrP}}K_{\text{NarR}}}}, \end{array}$$

(10) $$\begin{array}{l} f_{\text{act}}^{nir}=\frac{X_{\text{NNR}}}{X_{\text{NNR}}+K_{\text{NNR}}}, \end{array}$$

where *K*_FnrP_, *K*_NarR_ and *K*_NNR_ (all dimensionless) are the half-saturation constants for transcription factor binding to the operator site relative to the total transcription factor concentration.

Translation of mRNA into enzymes is described by first-order kinetics:

(11) $$\begin{array}{l} r_{\text{translation}}^{i}=k_{\text{translation}}^{i}T_{i}, \end{array}$$

in which *k*_translation_ [enzymes transcript^−1^ s^−1^] is the first-order translation coefficient and *T*_*i*_ [transcripts L^−1^] is the transcript concentration of gene *i*. Enzymes (*E*) and transcripts (*T*) undergo first-order decay with decay coefficients $k_{\text{dec}}^{T}$ [s^−1^] and $k_{\text{dec}}^{E}$ [s^−1^], respectively:

(12) $$\begin{array}{l} r_{\text{decay},\text{T}}^{i}=k_{\text{dec}}^{T}T_{i}, \end{array}$$

(13) $$\begin{array}{l} r_{\text{decay},\text{E}}^{i}=k_{\text{dec}}^{E}E_{i}. \end{array}$$

Dynamics of transcripts are usually fast, with transcript half-lives of only a few minutes (Härtig and Zumft, 1999; Bernstein et al., 2002), compared to enzyme half-lives and cell doubling times of several hours (Blaszczyk, 1993; Maier et al., 2011). Therefore, for transcript concentrations, we assumed a quasi-steady state (qss) (Ingalls, 2013):

(14) $$\begin{array}{l} \frac{\text{d}T_{i}}{\text{d}t}=r_{\text{transcription}}^{i}-r_{\text{decay},\text{T}}^{i}=0, \end{array}$$

which yields the quasi-steady state transcript concentrations $T_{i}^{\text{qss}}$ [transcripts L^−1^]:

(15) $$\begin{array}{l} T_{i}^{\text{qss}}=\beta_{T}^{i}f_{\text{act}}^{i}B, \end{array}$$

where $\beta_{T}^{i}=\alpha_{i}/k_{\text{dec}}^{T}$ is the number of transcripts per cell at maximum transcription, that is when $f_{\text{act}}^{i}$ equals 1. Enzyme concentrations are assumed to be governed by mRNA-translation as well as first-order decay:

(16) $$\begin{array}{l} \frac{\text{d}E_{i}}{\text{d}t}=r_{\text{translation}}^{i}-r_{\text{decay},\text{E}}^{i}. \end{array}$$

The translation rate constant is difficult to measure and the prior range of this parameter is therefore essentially unconstrained. However, we can express it in terms of parameters that are easier to estimate by computing the quasi-steady state (qss) of enzyme concentrations, $E_{i}^{\text{qss}}$ [enzymes L^−1^], given by

(17) $$\begin{array}{l} E_{i}^{\text{qss}}=\frac{k_{\text{translation}}^{i}\beta_{T}^{i}}{k_{\text{dec}}^{E}}f_{\text{act}}^{i}B. \end{array}$$

We defined $\beta_{E}=\frac{k_{\text{translation}}^{i}\beta_{T}^{i}}{k_{\text{dec}}^{E}}$ as the maximum quasi-steady state enzyme concentration per cell, a parameter that is easier to constrain based on literature data. We can then express the translation rate constant as

(18) $$\begin{array}{l} k_{\text{translation}}^{i}=k_{\text{dec}}^{E}\frac{\beta_{E}}{\beta_{T}^{i}}. \end{array}$$

The transformation rate of nitrogen substrate *j* (${\text{NO}}_{3}^{-}$ or ${\text{NO}}_{2}^{-}$) with corresponding enzyme *i* (NAR or NIR) is described by Michaelis–Menten kinetics considering kinetic inhibition of the reaction by O_2_:

(19) $$\begin{array}{l} r_{j}=r_{\text{max}}^{i}\frac{C_{j}}{C_{j}+K_{j}}\frac{I_{\text{reac}}^{i}}{I_{\text{reac}}^{i}+C_{{\text{O}}_{2}}}, \end{array}$$

in which the maximum rate constant $r_{\text{max}}^{i}$ [mol L^−1^ s^−1^] is the theoretical rate at full substrate saturation without inhibition. The second and third term of equation 19 describe substrate limitation and oxygen inhibition of the enzyme, in which *c*_*j*_ [M] is the concentration of substrate *j*, *K*_*j*_ is the half-saturation constant [M] of substrate *j* and $I_{\text{reac}}^{i}$ [M] is the oxygen-inhibition constant for the corresponding enzyme (*i*).

In a Monod-type model, the enzyme concentration inside a cell is assumed to be constant and *r*_max_ is proportional to the cell density:

(20) $$\begin{array}{l} r_{\text{max}}^{i}=\nu_{\text{max}}^{i}B, \end{array}$$

in which $\nu_{\text{max}}^{i}$ [mol cell^−1^ s^−1^] represents a constant, cell-specific maximum substrate-consumption rate. In the enzyme-based model, the transformation rate of substrate *i* is given by

(21) $$\begin{array}{l} r_{\text{max}}^{i}=k_{\text{max}}^{i}E_{i}, \end{array}$$

in which the turnover number $k_{\text{max}}^{i}$ [s^−1^] is the maximum amount of substrate that a single enzyme molecule can transform per unit time.

In both models (enzyme-based and Monod-type), aerobic respiration is described by a Monod rate law:

(22) $$\begin{array}{l} r_{{\text{O}}_{2}}=\nu_{\text{max}}^{{\text{O}}_{2}}B\frac{C_{{\text{O}}_{2}}}{C_{{\text{O}}_{2}}+K_{{\text{O}}_{2}}}, \end{array}$$

with the maximum cell-specific rate $\nu_{\text{max}}^{{\text{O}}_{2}}$ [mol cell^−1^ s^−1^] and half-saturation constant *K*_O_2__ [M]. *P. denitrificans* can utilize both aerobic respiration and denitrification for growth. However, typically reported growth yields for aerobic respiration are higher than those reported for denitrification (Boogerd et al., 1984) and, under the reported experimental conditions (Qu et al., 2015), growth using oxygen as the electron acceptor was the dominant process. Therefore, we assume that *P. denitrificans* only grows on aerobic respiration and that denitrification steps do not result in growth:

(23) $$\begin{array}{l} \frac{\text{d}B}{\text{d}t}=Y_{{\text{O}}_{2}}\frac{n_{{\text{C}}_{4}{\text{H}}_{6}{\text{O}}_{4}}}{n_{{\text{O}}_{2}}}r_{{\text{O}}_{2}}, \end{array}$$

in which *Y*_O_2__ [cells mol^−1^ of succinate] is the growth yield and *n*_C_4_H_6_O_4__ = 2 and *n*_O_2__ = 7 are the stoichiometric coefficients of succinate and oxygen in the energy reaction, respectively.

The flasks used in the experiment had a headspace. We accounted for the mass transfer rates $r_{\text{tr}}^{i}$ of gaseous compounds (N_2_ and oxygen) between the water and the gas phase. Furthermore, we also considered dilution of the gas phase by sampling. Details can be found in the Supplementary Material. The dynamics of solute concentrations of oxygen and nitrogen species are given by.

(24) $$\begin{array}{l} \frac{\text{d}C_{{\text{O}}_{2}}}{\text{d}t}=-r_{{\text{O}}_{2}}+r_{\text{tr}}^{{\text{O}}_{2}}, \end{array}$$

(25) $$\begin{array}{l} \frac{\text{d}C_{{\text{NO}}_{3}^{-}}}{\text{d}t}=-r_{{\text{NO}}_{3}^{-}}, \end{array}$$

(26) $$\begin{array}{l} \frac{\text{d}C_{{\text{NO}}_{2}^{-}}}{\text{d}t}=r_{{\text{NO}}_{3}^{-}}-r_{{\text{NO}}_{2}^{-}}, \end{array}$$

(27) $$\begin{array}{l} \frac{\text{d}C_{{\text{N}}_{2}}}{\text{d}t}=\frac{1}{2}r_{{\text{NO}}_{2}^{-}}+r_{\text{tr}}^{{\text{N}}_{2}}. \end{array}$$

### 2.3. Parameter Identification

A subset of the model parameters was fixed, either because these parameters were known from the experimental setup, or they could be well constrained by literature values. In total, we estimated 14 parameters of the Monod-type model and 32 parameters of the enzyme-based model with a Bayesian approach. Given the evidence of observed data and prior knowledge, we obtain the *posterior* probability distribution of parameter values. Following Bayes' law, the conditional probability density of parameters ***θ*** given the data ***y***, *p*(***θ***∣***y***), is proportional to the prior probability density *p*(***θ***) of the parameters and the likelihood of the measured data *p*(***y***|***θ***):

(28) $$\begin{array}{l} p\left(\theta |y\right)\propto p\left(y|\theta \right)p\left(\theta \right). \end{array}$$

The prior distribution constrains the parameters on knowledge uninformed by the data, such as literature values or physical constraints. The likelihood describes the probability density of the measured data if the model parameters were correct. For a perfect model, the likelihood only describes measurement errors, but in most applications it also include the effects of conceptual errors on meeting the data. We used the likelihood function

(29) $$\begin{array}{l} p\left(y|\theta \right)=\underset{i,j}{\prod }T\left(y_{ij}|\mu_{ij},\sigma_{ij},\nu \right), \end{array}$$

in which *T* is the Student's *t*-distribution with scale σ_*ij*_, degrees of freedom ν, and location

(30) $$\begin{array}{l} \mu_{ij}=\text{boxcox}\left(c_{ij}+b_{i},\lambda_{i}\right). \end{array}$$

The index *i* refers to the measured variable, *j* indicates the measurement number, and boxcox is the Box–Cox transform (Box and Cox, 1964). All measured data from the experiment were Box–Cox transformed to ensure homoscedasticity of the residuals. We added a constant background value *b*_*i*_ to the simulated concentration *c*_*ij*_ of oxygen and nitrite because measured concentrations never reached zero in the experiment, but stayed at a very low background value (<10^−7^ M for ${\text{NO}}_{2}^{-}$ and <2 × 10^−7^ M for O_2_). The errors σ_*ij*_ are the sum of data errors computed from the standard deviations of triplicate measurements and a constant error that accounts for model structural errors, which we chose to be an estimation parameter. We chose a Student's *t*-distribution with ν = 10 instead of a normal distribution so that outliers do not get too much weight.

The model was implemented in the programming language Python and the code is freely available (Störiko et al., 2021). We solved the system of ordinary differential equations (ODEs) using the Python package Sunode (Seyboldt, 2020), a Python wrapper to the CVODES library (Hindmarsh et al., 2005). Obtaining numerically stable results over a broad range of parameter values was difficult. Therefore, the ODE was log-transformed and solved using a backward differentiation formula of variable order (Byrne and Hindmarsh, 1975; Jackson and Sacks-Davis, 1980) with small tolerances (10^−12^ for the absolute and relative tolerance in the forward problem, 10^−8^ in the adjoint problem). Samples from the posterior distribution were drawn using the Python package PyMC3 (Salvatier et al., 2016) and analyzed with ArviZ (Kumar et al., 2019).

We sampled all models with the Hamiltonian MCMC algorithm No-U-Turn Sampler (NUTS; Hoffman and Gelman, 2014) as implemented in PyMC3, with adjusted settings for the tuning phase (Foreman-Mackey, 2020). It uses gradient information that we obtained by solving the adjoint sensitivity equations. We used the $\hat{R}$-criterion (Vehtari et al., 2020) that compares the variance between different chains to the in-chain-variance to assess convergence. The largest $\hat{R}$ is 1.005 for the Monod-type model and 1.021 for the enzyme-based model—the values close to one indicate convergence.

Supplementary Table 2 lists fixed parameter values, prior distributions, and statistics about the posterior distributions. Supplementary Table 1 provides initial values of the simulations.

Enzyme concentrations were not measured in the experiment and were only indirectly constrained through reaction rates. As all rate laws contain the product of enzyme concentrations and the maximum enzyme-specific turnover rate *k*_max_, it is impossible to estimate both *k*_max_ and β_*E*_ without direct measurements of enzyme concentrations. Therefore, we fixed β_*E*_ to an arbitrary value of 1125 enzymes cell^−1^ based on measured intracellular enzyme concentrations in the literature (Maier et al., 2011). This implies that the fitted values of *k*_max_ and the simulated enzyme concentrations within bacterial cells have to be interpreted with care, as they are conditioned on the arbitrary choice of β_*E*_.

## 3. Results and Discussion

### 3.1. Modeled Concentration Time Series

**Figure 2:**
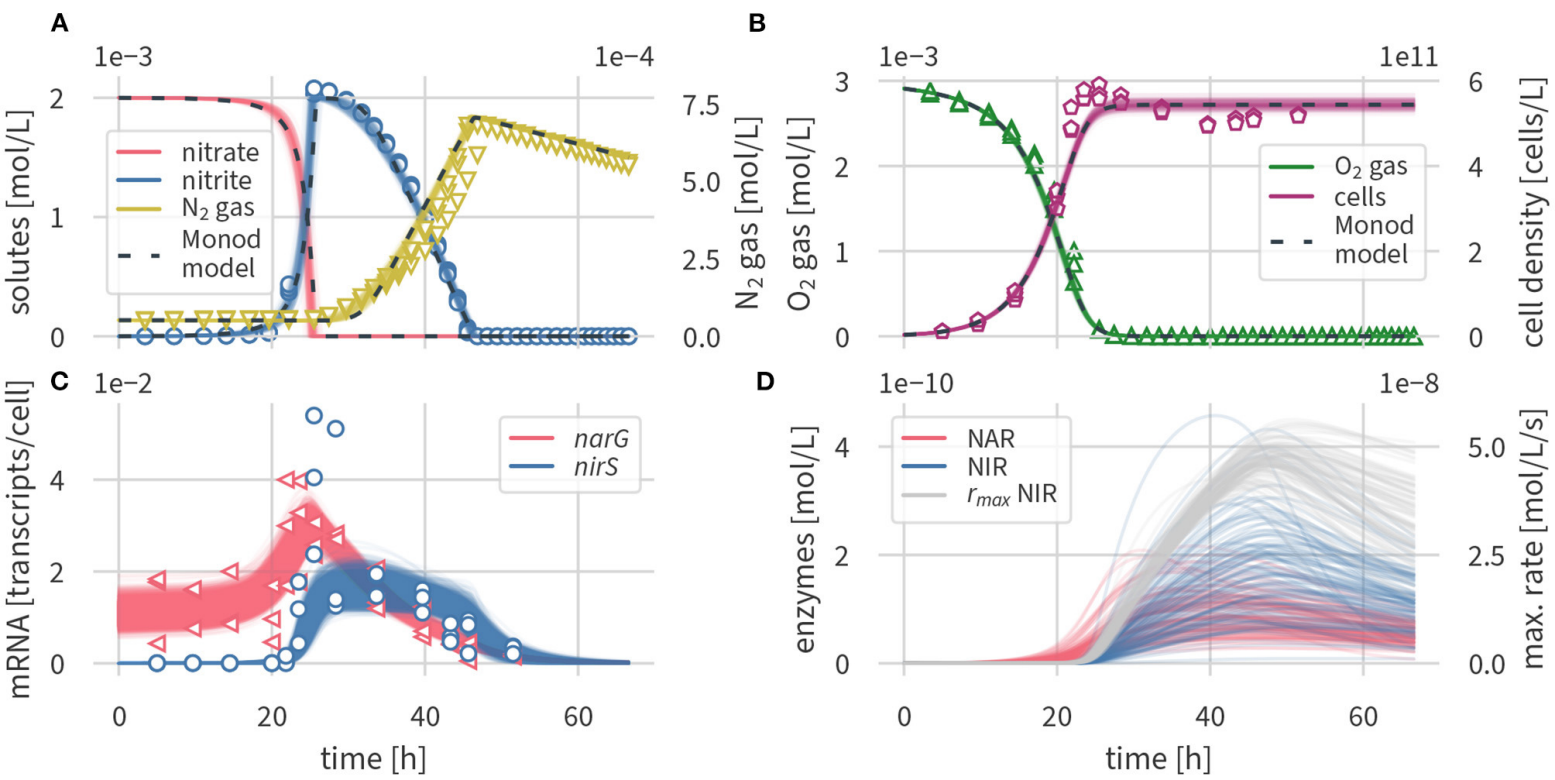
Simulated dynamics, 100 draws from the posterior of the enzyme-based model (solid lines) and posterior median of the Monod-type model (dashed lines), and measurements from three experimental replicates (open symbols). **(A)** Nitrogen compounds, **(B)** oxygen and cell densities, **(C)** transcripts, and **(D)** enzymes and maximum rates.

Figure 2 shows a comparison of the simulated concentrations of the enzyme-based and the Monod-type models with the experimental data (Qu et al., 2015). Figure 2A shows the concentrations of nitrogen species (nitrate, nitrite, N_2_) of the models and the experiments, Figure 2B shows the oxygen concentrations and cell densities, Figure 2C shows the transcripts of *narG* and *nirS*, respectively, whereas Figure 2D contains simulated enzyme concentrations for which no measurements were available. Fitted parameter values and their uncertainties are outlined in Supplementary Table 2, and simulated transcription factor concentrations are shown in Supplementary Figure 4.

Concentration times series results in Figures 2A,B are present as several draws of the posterior distribution for the enzyme based model and the median output for the Monod-type model. Results for the Monod-type model exhibited a similarly narrow spread of simulated concentrations, and thus multiple draws were omitted. Both model formulations fit the concentration data equally well. The excellent fit supports the validity of our conceptual model and the underlying assumptions outlined above. Oxygen is consumed before nitrate and nitrite are reduced to molecular nitrogen, which confirms the inhibition of denitrification by oxygen. The simulations predict that nitrate is fully converted to nitrite before the onset of the second denitrification step. Both models capture the sharp increase in nitrite concentrations, peaking after 25 h at 2 mM, followed by a gradual decrease over 40 h, and the subsequent production of N_2_ gas, which is the final product of denitrification. The posterior distributions of solute and gas concentrations in both models were narrow, reflecting the small measurement variance. Even though nitrate concentrations were not measured by Qu et al. (2015), the nitrate simulation results are indirectly well constrained by nitrite data and the mass conservation assumption encoded in the model equations.

At the time of highest nitrite concentrations, the measured cell densities reached peak values and gradually decreased (Figure 2B). Neither of the models was able to capture the slightly non-monotonic behavior in cell densities with a peak at 25 h and a subsequent modest decline. The slight discrepancy between simulated and measured cell densities could be due to several assumptions of our model. We assumed that *P. denitrificans* only grows on oxygen, even though the organism is known to grow on both oxygen and nitrate (Boogerd et al., 1984), which may explain growth beyond the time of oxygen availability. The subsequent slight decrease in cell densities, if not a measurement artifact, may be a toxicity effect of nitrite or one of the non-modeled intermediates. Nitrite is known to be toxic to *P. denitrificans* and other microorganisms at millimolar concentrations (van Verseveld et al., 1977; Stouthamer, 1980; Zhang et al., 2013). Accounting for nitrite toxicity has been postulated as an explanation for delayed growth and increased cell lysis of *Shewanella oneidensis* while reducing nitrite to ammonium (Mellage et al., 2019). Altogether, we deemed the misfit in cell densities not significant enough to justify making the model more complex by introducing additional processes with difficult-to-constrain parameters.

### 3.2. Transcript and Enzyme Dynamics

Overall, the enzyme-based model formulation captured the dynamics of measured *narG* and *nirS* concentrations (Figure 2C). Both simulated and measured *narG* transcript concentrations fluctuated around a baseline value of 10^−2^ transcripts cell^−1^, and *nirS* levels were very low during the oxic phase. Following a drop in oxygen levels, simulated *nar* transcripts rose, followed by those of *nir*. The model satisfactorily captured the peak of *narG* transcripts at 23 h and the following gradual decrease to low, but detectable transcript levels at the last measurement time point, at about 50 h. The model also predicted the very abrupt increase of *nir* transcripts at 24 h. While peak levels of measured *nir* transcripts at 25 h could not be matched, the measurement uncertainty of the transcript data at that time point was very large (2.4 × 10^−2^ transcripts cell^−1^ to 5.4 × 10^−2^ transcripts cell^−1^), and thus we considered these peak values as outliers. Our assumption is supported by additional data of a parallel experiment with the same setup, but using butyrate instead of succinate as the carbon source, where a peak in *nir* transcripts was not detected (Qu et al., 2015). Analogous to measured data, both of the simulated transcripts remained at concentrations above the limit of detection for a few hours after complete denitrification.

As seen in Figure 2D, enzyme concentrations were subject to considerable uncertainty—an expected result, as there are no data to constrain them. However, the maximum cell-specific turnover rate of NIR $r_{\text{max}}^{\text{NIR}}$, given by the product of enzyme concentrations and the maximum enzyme specific turnover rate, was well constrained, provided that substrate was being consumed. The solute data that we used for conditioning could apparently provide sufficient information about reaction rates to estimate the maximum rate.

We also set up a model version with a more simplified description of transcriptional regulation, for comparison. The model, described in the Supplementary Material, yielded similar results (Supplementary Figure 6) to those outlined in Figure 2. However, the simplified model formulation was unable to capture the sudden increase in *nirS* transcripts (at *t* = 24 h). Therefore, we opted for the more complex transcription factor based formulation.

### 3.3. Posterior Parameter Uncertainty

**Figure 3:**
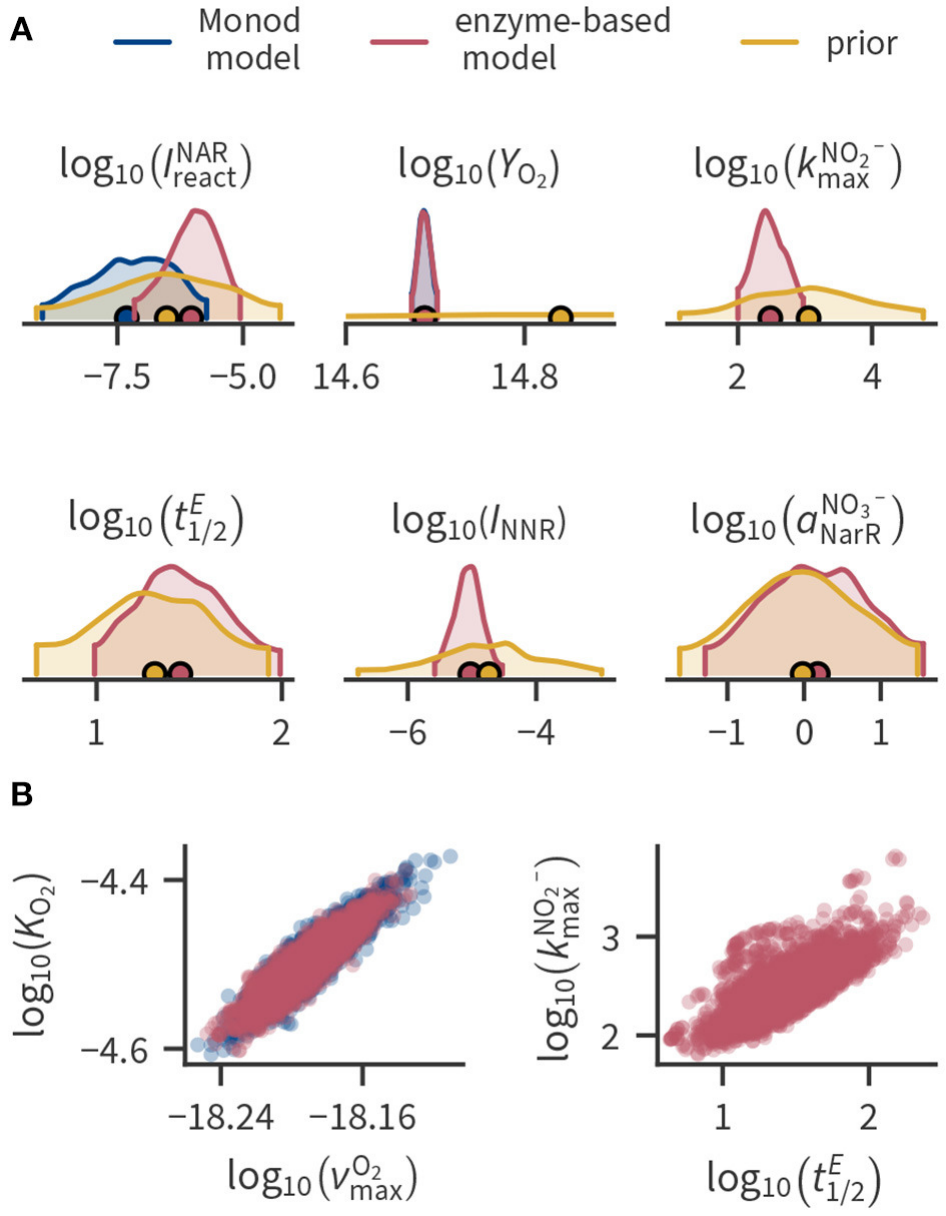
Selected parameter distributions. **(A)** Kernel density estimates of the marginal distributions of selected parameters (marginal distributions of all parameters can be found in the Supplementary Material). Densities are cut off at the 94 % highest density intervals, circular markers indicate the mean. Parameters $k_{\text{max}}^{{\text{NO}}_{2}^{-}}$, $t_{1/2}^{E}$, *I*_NNR_ and $a_{\text{NarR}}^{{\text{NO}}_{3}^{-}}$ are not present in the Monod-type model such that the posterior is only shown for the enzyme-based formulation. Posterior distributions of *Y*_O_2__ are similar in both model formulations and cannot be distinguished in the plot. **(B)** Pairwise joint posterior distributions of parameters with strong correlation.

Figure 3 shows prior and posterior distributions of selected parameters. In our application, most posterior parameter distributions were much narrower than the prior ranges (Figure 3A), indicating that they were strongly constrained by the available data (e.g., *Y*_O_2__). Where applicable, the Monod-type and enzyme-based models mostly resulted in similar parameter distributions. However, the estimated enzyme inhibition constants $I_{\text{reac}}^{\text{NAR}}$ and $I_{\text{reac}}^{\text{NIR}}$ were smaller in the Monod-type model compared to the enzyme-based model because the Monod-type model compensates the lacking regulation of enzyme levels with a stronger inhibition of enzymes by oxygen. Some parameters exhibit considerable spread even after conditioning to the data, partly ranging over several orders of magnitude. This holds mainly for parameters that are related to transcription factor, transcript and enzyme concentrations such as transcription factor activation rate constants (e.g., $a_{\text{NarR}}^{{\text{NO}}_{3}^{-}}$) or the enzyme half-life $t_{1/2}^{E}$.

In some cases, part of the spread can be attributed to the correlation among the conditional parameters, as shown in Figure 3B for selected examples. Supplementary Figures 4, 5 of the Supplementary Material contain all correlation coefficients of the posterior distributions. Figure 3B illustrates that the enzyme half-life $t_{1/2}^{E}$ strongly correlates with the “efficiency” of NIR enzymes expressed by $k_{\text{max}}^{{\text{NO}}_{2}^{-}}$. The enzyme half-life influences the concentration of the enzymes. Because the total reaction rate depends on the product of the enzyme concentration with the efficiency of a single enzyme, changing the enzyme half-life can be compensated by also changing the enzyme efficiency. Thus, if we had enzyme data, we could significantly decrease the uncertainty in enzyme half-lives and the enzyme-specific maximum turnover rate $k_{\text{max}}^{{\text{NO}}_{2}^{-}}$ of nitrite.

A strong correlation can also be observed between the Monod parameters related to aerobic respiration (Figure 3B), which has been previously reported (Liu and Zachara, 2001). Reparameterization alleviated the correlation between $\nu_{\text{max}}^{{\text{O}}_{2}}$ and *K*_O_2__. The parameter $\nu_{\text{max}}^{{\text{O}}_{2}}$ is the cell-specific respiration rate at the limit of the substrate concentration approaching infinity. Instead, we used the cell-specific respiration rate at a fixed concentration $\nu_{\text{fix}}^{{\text{O}}_{2}}$ as a parameter (see Section 4 in the Supplementary Material). $\nu_{\text{fix}}^{{\text{O}}_{2}}$ and *K*_O_2__ were less correlated than before the reparametrization, but the correlation of $\nu_{\text{fix}}^{{\text{O}}_{2}}$ with the growth yield was larger.

### 3.4. Relationship Between Reaction Rates and Transcript Concentrations

**Figure 4:**
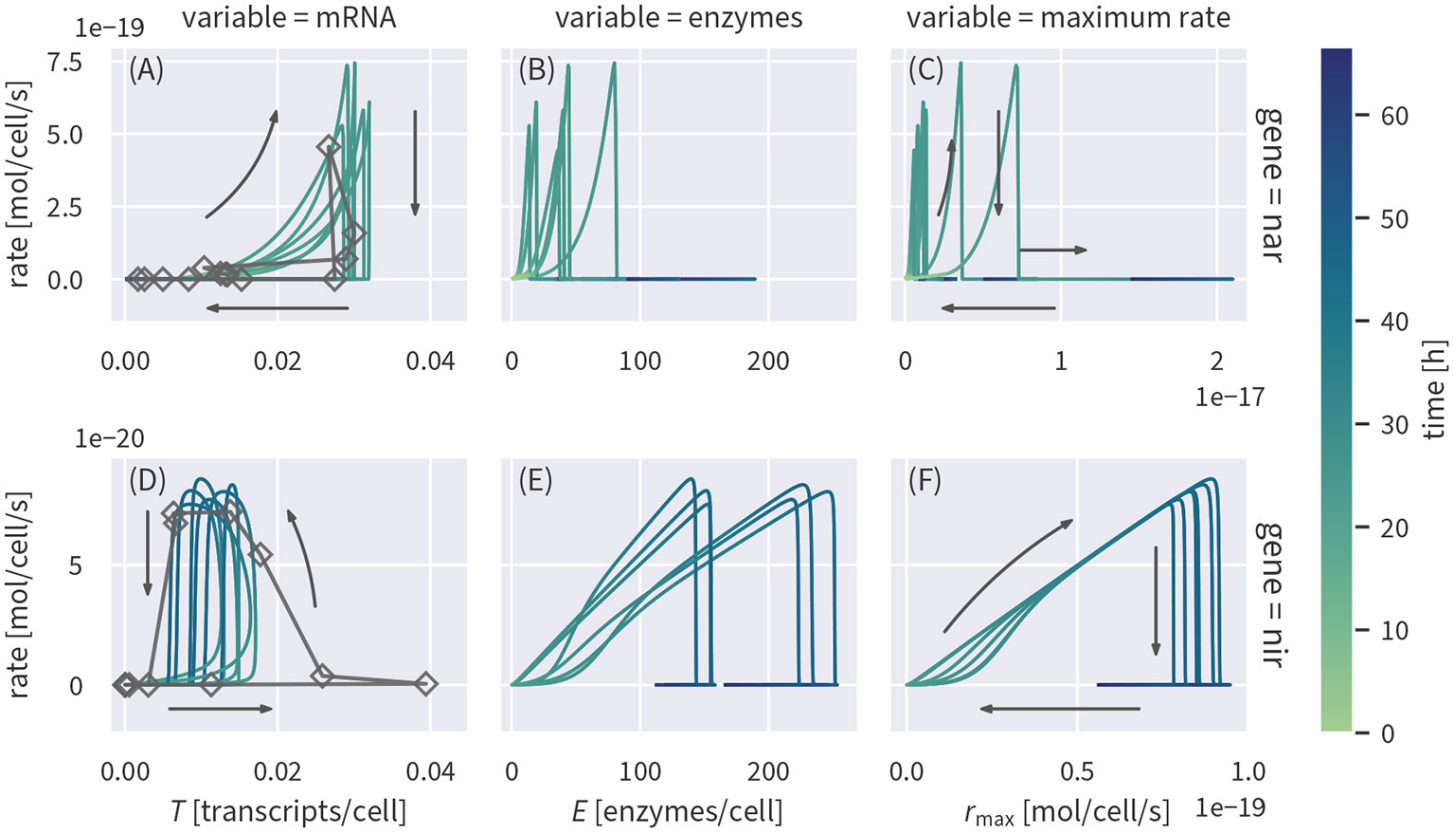
Cell-specific substrate turnover rates plotted against concentrations of the corresponding transcripts **(A,D)** or enzymes **(B,E)** and the maximum specific rate **(C,F)** linked to the reaction. The plot shows several draws from the posterior (colored lines). Measured transcript data are plotted against rates based on the Monod model (dark gray lines and rectangles).

It has been previously suggested to use transcript concentrations as a proxy for reaction rates (Nicolaisen et al., 2008; Freitag and Prosser, 2009; Brow et al., 2013; Achermann et al., 2020; Rohe et al., 2020). To test if such a relationship would be a valid assumption, we plot the simulated transcript concentrations and corresponding reaction rates against each other in Figures 4A,D. Cell-specific rates were calculated by applying the rate laws to the simulated concentrations and normalizing the result by the cell densities. Measured transcript concentrations plotted against the reaction rates based on the Monod-type model are shown for comparison.

Transcript concentrations of *nar* and *nir* are upregulated before rates increase (Figures 4A,D). Between 10 h to 25 h, *nar* transcripts show a positive, but non-linear correlation with reaction rates, before the rates drop to zero once nitrate is depleted. Nitrite reduction rates, in contrast, only start increasing when *nir* transcript levels have nearly reached their maximum (Figure 4D). Rates continue to increase when *nir* transcript levels start to drop such that rates and transcript levels are anti-correlated around 40 h, just before reaction rates drop abruptly upon substrate depletion. After the rapid drop in reaction rates, both transcripts remain present for several hours. We also looked at the relationship of enzyme concentrations with denitrification rates (Figures 4B,E). Enzyme concentrations are not well constrained by the data and their posterior uncertainty propagates to the relationship with reaction rates. We therefore plotted the product of enzyme concentrations and their “efficiency” $k_{\text{max}}^{{\text{NO}}_{2}^{-}}$, corresponding to the maximum potential reaction rate, without accounting for enzyme inhibition or substrate limitation (Figures 4C,F). The maximum rate of NIR is well constrained and shows an almost linear relationship with actual reaction rates up to the point where nitrite depletion cuts the rate (Figure 4F). The rate of nitrite reduction is essentially not limited by oxygen inhibition or substrate limitation. The maximum rate is thus the determining factor such that an increase in enzyme concentrations directly increases the rate. The relationship of NAR maximum rates and actual nitrate reduction rates (Figure 4C) is more complicated. The initial increase is strongly non-linear and the remaining posterior spread of the maximum rate leads to uncertainty in the scaling factor. Both findings can be explained by the initial inhibition of NAR enzymes by oxygen: NAR enzymes are produced while oxygen is still present so that *r*_max_ increases, but the actual rate does not, driven by the weaker inhibition of *enzyme production* relative to that of the *enzymatic catalysis*. The posterior uncertainty of the relationship is due to correlation between the oxygen inhibition parameter and the maximum rate. Different combinations of the parameters can yield the same reaction rate and an unambiguous assignment is not possible for a case when the data only constrain the effective rate.

Our results show that under the dynamic conditions of the experiment, transcript concentrations are not a good predictor of reaction rates. The complicated relationships are mainly caused by the asynchronism and different time scales of substrate dynamics, transcript and enzyme production, and decay. Under environmental conditions, changes in concentrations of redox-active species may be much slower than in the laboratory experiment simulated in this study. In groundwater, for example, the typical time scales are on the order of several months (Arora et al., 2013, 2016). If changes in redox-active species are slower than the enzyme dynamics (acting on the order of days), we would expect a better correlation between transcript and enzyme concentrations and reaction rates, potentially allowing for further simplifications of model formulations. However, many environments in which nitrogen cycling plays a crucial role are very dynamic. For example, rapid water-content driven changes in the redox state of soils (Pronk et al., 2020) or diurnal fluctuations in river biogeochemistry (Kunz et al., 2017) can control nitrogen turnover. The lack of obvious correlations between transcripts of functional genes and reaction rates observed in our modeling results are likely representative for such dynamic environments. A non-linear, hysteretic relationship between transcript concentrations and reaction rates has also been found in a gene-centric model of pesticide degradation (Chavez Rodriguez et al., 2020). Interestingly, this is the case although pesticide degradation acts on much longer time scales than denitrification in this study and the observed relationship between rates and transcripts shows different patterns than the ones presented in Figure 4 (e.g., direction of the hysteresis). This highlights the need to assess the relationship between gene expression and rates for each reaction system individually. Nevertheless, models that explicitly simulate transcript concentrations, as presented in this study, can help to understand the relationship between gene expression and rates and extrapolate it.

**Figure 5:**
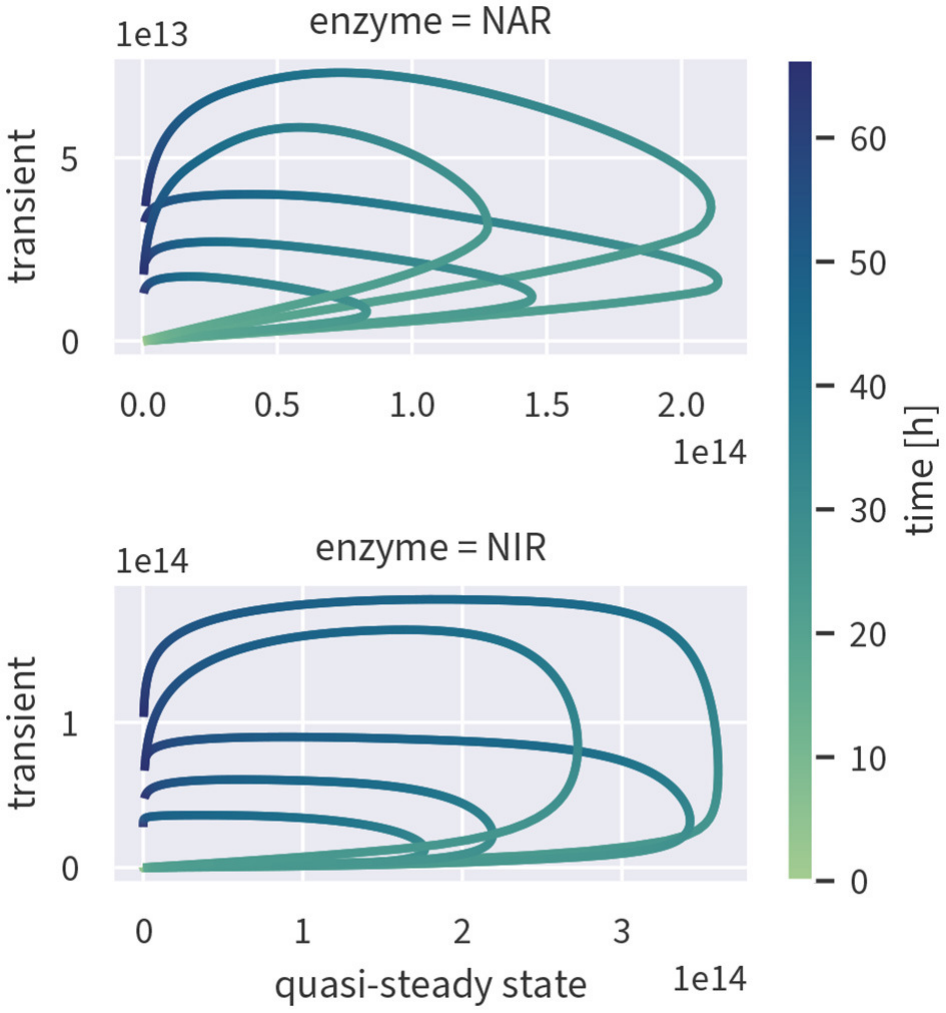
Quasi-steady enzyme state concentrations [enzymes L^−1^] plotted against the concentrations simulated by the fully transient model. Although the exact relationship varies between different draws from the posterior (indicated by several lines), all of them are characterized by hysteresis.

If enzyme concentrations were at quasi-steady state, the enzyme-based model could be simplified, reducing the number of state variables and parameters. To test if this assumption is valid, we ran the fully transient model version as shown in Figure 2) and computed the quasi-steady state concentrations based on the fully transient results. If the quasi-steady state assumption were valid, the two concentrations should cluster along the 1:1-line. As evident from Figure 5, this is not the case—enzyme concentrations exhibit a strongly hysteretic relationship and remain much lower than the corresponding quasi-steady state concentrations. Enzyme dynamics, as measured by estimated enzyme half-lives, are considerably slower than substrate dynamics such that the substrate stimulus is too short for enzyme concentrations to reach their quasi-steady state value.

### 3.5. Overall Model Performance

Our parsimonious enzyme-based model describes expressional patterns well and simulates denitrification rates very accurately. The model quantitatively links concentrations of functional transcripts to turnover rates. In reproducing the solute-concentration data of Qu et al. (2015), however, the Monod-type model is as good as the enzyme-based model. Small data errors lead to narrow posterior distributions of the solute concentrations in both models, whereas the much more uncertain transcript data do not help to further constrain them. While the enzyme-based model can be calibrated using more types of data than the Monod-type model, it also requires estimating more parameters, which appear to be weakly constrained. The strength of the enzyme-based model lies in improving our mechanistic understanding in a quantitative way. For example, it helps to decompose the contributions of transcriptional regulation and enzyme inhibition to the down-regulation of denitrification rates by oxygen. It confirms that the conceptual model of transcriptional regulation of *narG* and *nirS* through FnrP, NarR, and NNR can also explain expressional patterns quantitatively. Finally, the quantitative model helps to understand the non-linear relationship between rates and transcript or enzyme concentrations. It can be used to generate hypotheses about the behavior in other systems and develop sampling strategies for these quantities.

Transcription-factor regulation and enzyme dynamics need to be explicitly described as these processes take place at similar or even larger time scales than the actual enzymatic reactions and the corresponding substrate dynamics. The relation between transcript concentrations and reaction rates exhibits a strong hysteresis. Enzyme concentrations show a better correlation with reaction rates at early times but are uncorrelated at later times due to substrate limitations. Thus, enzyme kinetics and the abundance of enzymes present likewise have an impact on reaction rates and need to be taken into account.

### 3.6. Transferability to Environmental Systems

While the experimental conditions during the experiment we simulated are not representative for many environments (high succinate concentrations, single organism), the controlled conditions allowed us to set up a detailed and mechanistic model of the denitrification reactions. The presented model equations hereafter can be integrated in a model that better represents natural environments, for example, by accounting for transport or carbon limitation. Representing all denitrifiers by a single organism in the model certainly does not account for all interactions that occur in natural microbial communities. Considering a single pool of denitrifiers in quantitative reaction models, however, is a common practice to keep the simulations computationally and conceptually tractable (Kinzelbach et al., 1991; Sanz-Prat et al., 2015; Yan et al., 2016). In addition, *P. denitrificans* is a denitrifying organism that is actually present in natural environments like soils (Nokhal and Schlegel, 1983). The exact mechanism of transcriptional regulation is not the same in all denitrifiers, but some basic principles are similar in various studied organisms (Gaimster et al., 2018). Further, the exact pattern of gene expressional dynamics does not matter for reaction rates because they are smoothed out by the much slower enzyme dynamics. A simplified representation of the transcriptional regulation as we present in the Supplementary Material might describe the behavior of a diverse microbial community sufficiently well, where the effective expressional patterns would be some smoothed average of the individual patterns produced by different regulation mechanisms.

An alternative approach to modeling transcriptional regulation that does not require exact knowledge of the underlying mechanism has been presented previously by Song et al. (2017). The latter authors applied a cybernetic approach (Song and Liu, 2015) that relies on the principle of return-on-investment: Microorganisms will transcribe those genes that maximize their metabolic “profits” (though it is often unclear how to express that in quantitative terms). Compared to the simulation results of the enzyme-based denitrification model presented earlier (Song et al., 2017), we can observe some key differences. While transcript concentrations respond relatively quickly to changes in substrate concentrations, Song et al. (2017) predict a long delay in the response of transcripts. At the same time, enzyme concentrations respond to transcripts quickly in their model. While this is one possibility to explain the metabolic lag they observed, it is not necessary to ascribe the delay to slow *transcription* dynamics. Transcript dynamics in their model are not constrained by data so the actual lag could also be due to delayed *translation*. Differences between the model outcomes might be attributed to the different environmental systems. The laboratory system that we simulated exhibited faster kinetics and was more dynamic than the sediment that Song et al. (2017) analyzed (complete denitrification within 2 days with the pronounced accumulation of reactive intermediates, compared to a week). In contrast to the aforementioned study (Song et al., 2017), our simulations and transcript data support an immediate transcriptional response to denitrifying conditions, justifying the omission of a resource pool which first builds up before transcription starts in the model formulation.

The authors of the experimental data used here measured biomass in cell densities, which we also chose as state variable. In the natural environment, functional biomass is difficult to measure and a different approach is needed. A gene-centric approach that uses gene concentrations as proxies for functional biomass (Reed et al., 2014; Pagel et al., 2016) could be readily integrated into our model. Gene-centric approaches have been mostly applied to marine environments and their application to soils or groundwater might need extensions to account for relic DNA, which has been shown to represent as much as 40 % (Carini et al., 2016).

### 3.7. Implications for Biogeochemical Modeling

We informed our model with highly resolved time series of all solute concentrations in a controlled experiment. Under these conditions, the quantitative prediction of reaction rates was not improved by integrating transcript data. Since substrate and product concentrations are easier to obtain than transcript concentrations, quantitative rate predictions can be more effectively improved by additional chemical measurements than by integrating transcript concentrations. By contrast, transcript data can support the identification of key reactions, particularly if intermediate products have low concentrations and cannot be detected. In addition, transcript-based models can fill a key gap in our predictive capabilities within natural systems where the difficulty in acquiring cell density information impedes the validation of biomass-explicit models.

The advancement of enzyme-based models requires appropriate data for evaluating models and testing hypotheses. Datasets with quantitative information about functional genes, transcripts *and* enzymes could help to find accurate and parsimonious descriptions of transcript and enzyme dynamics. Measuring enzyme concentrations (e.g., by targeted quantification of functional enzymes, Li et al., 2017b) seems more promising than transcript concentrations as they more closely relate to actual turnover rates. However, measuring specific proteins in environmental samples may be restricted by major challenges, such as the efficient extraction of proteins from environmental samples, hindering accurate quantification and limiting the wider application of functional enzyme measurements (Starke et al., 2019).

We are convinced that further improvements of enzyme-based models in environmental systems can be achieved by integrating data from experiments carried out under different environmental conditions, expanding the sensitivity range of simulated processes and parameters. While batch experiments with high cell densities and nutrient concentrations usually exhibit fast dynamics there is a need to condition models to data from less dynamic systems such as chemostats and flow-through columns, which may more closely relate to natural environmental conditions.

## Data Availability Statement

The datasets presented in this study can be found in online repositories. The names of the repository/repositories and accession number(s) can be found below: www.doi.org/10.5281/zenodo.4620167.

## Author Contributions

AS developed and implemented the models, and prepared the original draft, supervised by HP and OC. OC was responsible for funding acquisition. AS, HP, AM, and OC contributed to conceptualization, visualization, study design, writing, and revisions of the original draft. All authors contributed to the article and approved the submitted version.

## Conflict of Interest

The authors declare that the research was conducted in the absence of any commercial or financial relationships that could be construed as a potential conflict of interest.
