# Supplementary material for: Does It Pay Off to Explicitly Link Functional Gene Expression to Denitrification Rates in Reaction Models?
Source: Front Microbiol. 2021 Jun 18;12:684146. doi: 10.3389/fmicb.2021.684146 (PMC8250433; doi:10.3389/fmicb.2021.684146)
Supplement: Supplementary file 1 [file Presentation_1.PDF]

Supplementary Material

Does It Pay Off to Explicitly Link Functional Gene Expression to Denitrification Rates in Reaction Models?

Anna Störiko[1], Holger Pagel[2], Adrian Mellage[1], and Olaf A. Cirpka[1]

[1]*Center for Applied Geoscience, University of Tübingen, Tübingen, Germany*
[2]*Biogeophysics, Institute of Soil Science and Land Evaluation, University of Hohenheim, Stuttgart, Germany*

Contents

1 Initial Values S1
2 Prior and Posterior Parameter Distributions S2
3 Transcription Factor Concentrations S8
4 Reparametrization of Monod Parameters S8
5 Mass Transfer to the Gas Phase and Gas Sampling S9
6 Simplified Model of Transcriptional Regulation S10

1 Initial Values

Table S1 lists the initial values of all solute and gas concentrations. Transcription factor concentrations are initialized based on their quasi-steady state concentrations and, thus, depend on the values of the regulation parameters.

Table S1: Initial concentration values used for the simulation.

Substance	Value	Units	Reference
Nitrate	2×10^{-3}	M	known from experimental set-up
Nitrite	0	M	known from experimental set-up
N_2 in the gas phase	5.2×10^{-5}	M	measurement data
N_2 in water	8.7×10^{-7}	M	equilibrium with the gas phase
O_2 in the gas phase	2.9×10^{-3}	M	known from experimental set-up
O_2 in water	9.3×10^{-5}	M	equilibrium with the gas phase
Cells	variable	cells L^{-1}	estimation parameter (see table S2)
active fraction of FnrP/NarR/NNR	variable	dimensionless	quasi-steady state concentration
NAR/NIR enzymes	0	M	assumption

2 Prior and Posterior Parameter Distributions

Figure S1 shows the prior and posterior distributions of all parameters related to the ordinary differential equations (ODEs) used in the Monod-type and enzyme based model. Table S2 lists the values of fixed parameters and the prior distributions of the fitted parameters. All parameters related to transcript and enzyme concentrations were estimated with the exception of the maximum enzyme concentration β_E. Figures S2 and S3 show pairwise correlation coefficients of all parameters in the Monod-type and enzyme-based models, respectively.

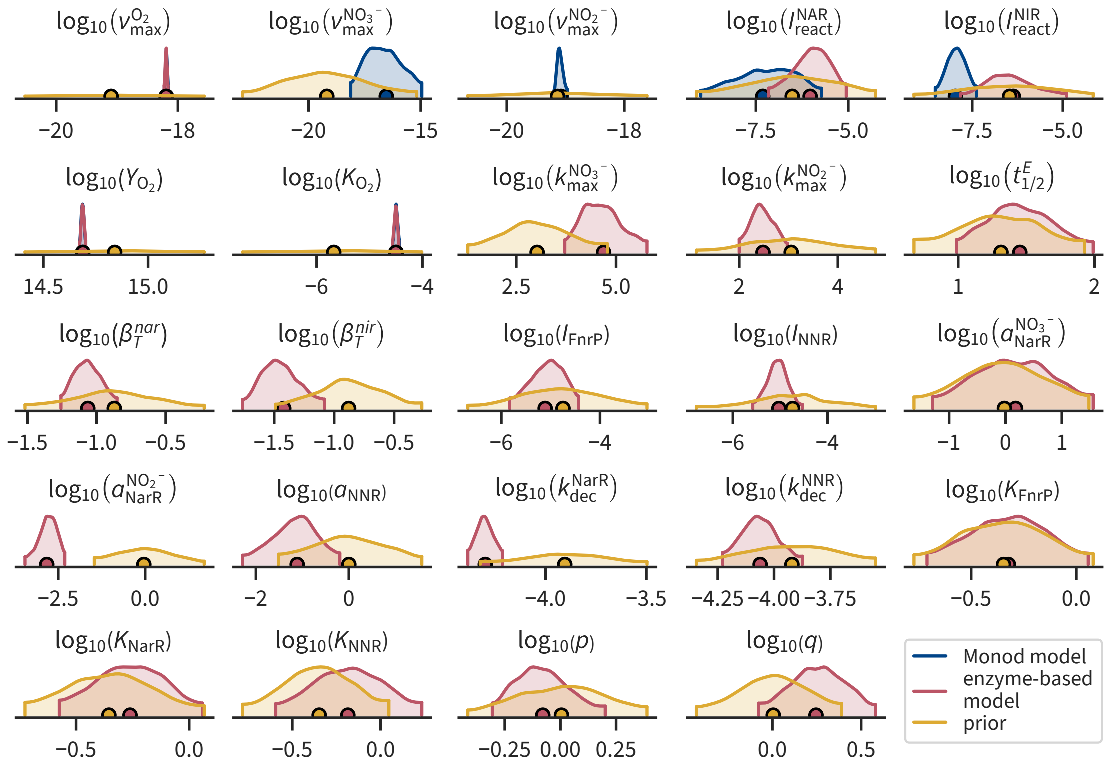

Figure S1: Kernel density estimates of the marginal posterior and prior distributions for all model parameters. Densities are cut off at the 94 % highest density intervals, circular markers indicate the mean.

Table S2: Simulation parameters, their prior distributions or fixed values, and their posterior medians and percentiles.

Symbol	Description	Units	Prior[a]/Value	Reference	Posterior Percentiles 10 %	Median	90 %	Model[b]
$t^E_{1/2}$	enzyme half life	h	$N(3, 0.8)$	f, g	1.3×10^1	2.8×10^1	6.4×10^1	E
β_T^{nar}	maximum *narG* concentration	transcripts cell^{-1}	$T(-2, 0.7, 10)$	f, h	6.3×10^{-2}	8.5×10^{-2}	1.2×10^{-1}	E
β_T^{nir}	maximum *nirS* concentration	transcripts cell^{-1}	$T(-2, 0.7, 10)$	f, h	2.3×10^{-2}	3.5×10^{-2}	6.7×10^{-2}	E
$I_{\mathrm{trs}}^{\mathrm{FnrP}}$	O_2 inhibition constant of FnrP	M	$T(-11, 2, 10)$	c	2.3×10^{-6}	8.6×10^{-6}	2.2×10^{-5}	E
$I_{\mathrm{trs}}^{\mathrm{NNR}}$	O_2 inhibition constant of NNR	M	$T(-11, 2, 10)$	c	4.4×10^{-6}	8.9×10^{-6}	1.8×10^{-5}	E
p	Hill coefficient for FnrP	–	$N(0, 0.5)$		5.7×10^{-1}	8.2×10^{-1}	1.2	E
q	Hill coefficient for NNR	–	$N(0, 0.5)$		1.0	1.8	2.9	E
$a_{\mathrm{NarR}}^{\mathrm{NO_3^-}}$	NarR activation constant for NO_3^-	s^{-1} M^{-1}	$N(0, 2)$	c	1.6×10^{-1}	1.4	1.5×10^1	E
$a_{\mathrm{NarR}}^{\mathrm{NO_2^-}}$	NarR activation constant for NO_2^-	s^{-1} M^{-1}	$N(0, 2)$	c	5.6×10^{-4}	1.6×10^{-3}	3.4×10^{-3}	E
a_{NNR}	NNR activation constant for NO_2^-	s^{-1} M^{-1}	$N(0, 2)$	c	1.4×10^{-2}	7.7×10^{-2}	3.8×10^{-1}	E
$k_{\mathrm{dec}}^{\mathrm{NarR}}$	dissociation constant of NarR	s^{-1}	$N(-9, 0.5)$		4.4×10^{-5}	5.0×10^{-5}	5.8×10^{-5}	E
$k_{\mathrm{dec}}^{\mathrm{NNR}}$	dissociation constant of NNR	s^{-1}	$N(-9, 0.5)$	k	6.7×10^{-5}	8.6×10^{-5}	1.1×10^{-4}	E
K_{FnrP}	half-saturation constant for binding of FnrP	–	$N(-0.8, 0.5)$		2.5×10^{-1}	4.8×10^{-1}	8.7×10^{-1}	E
K_{NarR}	half-saturation constant for binding of NarR	–	$N(-0.8, 0.5)$		3.3×10^{-1}	5.5×10^{-1}	9.0×10^{-1}	E
K_{NNR}	half-saturation constant for binding of NNR	–	$N(-0.8, 0.5)$		3.4×10^{-1}	6.6×10^{-1}	1.3	E
$I_{\mathrm{reac}}^{\mathrm{NAR}}$	O_2 inhibition parameter for reaction of NO_3^-	M	$N(-15, 3)$		3.0×10^{-9}	5.9×10^{-8}	6.1×10^{-7}	M
					1.6×10^{-7}	1.0×10^{-6}	4.3×10^{-6}	E
$I_{\mathrm{reac}}^{\mathrm{NIR}}$	O_2 inhibition parameter for reaction of NO_2^-	M	$N(-15, 3)$		4.6×10^{-9}	1.1×10^{-8}	2.6×10^{-8}	M
					6.1×10^{-8}	3.4×10^{-7}	4.4×10^{-6}	E

Continued on the next page.

Table S2: (continued) Simulation parameters, their prior distributions or fixed values, and their posterior medians and percentiles.

Symbol	Description	Units	Prior[a]/Value	Reference	Posterior Percentiles 10 %	Median	90 %	Model[b]
K_{O_2}	O_2 half-saturation constant	M	$T(-13, 2, 10)$	i	2.9×10^{-5}	3.2×10^{-5}	3.5×10^{-5}	M
					2.9×10^{-5}	3.1×10^{-5}	3.4×10^{-5}	E
$K_{NO_3^-}$	NO_3^- half-saturation constant	M	5×10^{-6}	j		fixed value		E, M
$K_{NO_2^-}$	NO_2^- half-saturation constant	M	5×10^{-6}	j		fixed value		E, M
Y_{O_2}	O_2 growth yield per amount e^--donor	cells mol^{-1}	$T(32.2, 0.5, 10)$	j	4.8×10^{14}	4.9×10^{14}	5.0×10^{14}	M
					4.8×10^{14}	4.9×10^{14}	5.0×10^{14}	E
$v_{fix}^{O_2}$	O_2 reaction rate at fixed O_2 concentration	mol cell^{-1} s^{-1}	$T(-44.2, 1.6, 10)$	d	4.1×10^{-19}	4.2×10^{-19}	4.3×10^{-19}	M
					4.1×10^{-19}	4.2×10^{-19}	4.3×10^{-19}	E
$v_{fix}^{NO_2^-}$	NO_2^- reaction rate at fixed O_2 concentration	mol cell^{-1} s^{-1}	$T(-44.2, 1.7, 10)$	d	2.3×10^{-22}	4.8×10^{-22}	1.1×10^{-21}	M
$v_{fix}^{NO_3^-}$	NO_3^- reaction rate at fixed O_2 concentration	mol cell^{-1} s^{-1}	$T(-49.4, 3.3, 10)$	d	1.7×10^{-20}	7.1×10^{-20}	5.0×10^{-19}	M
$k_{max}^{NO_3^-}$	NAR turnover number	s^{-1}	$T(7, 2, 10)$		7.5×10^{4}	2.5×10^{5}	1.4×10^{6}	E
$k_{max}^{NO_2^-}$	NIR turnover number	s^{-1}	$T(7, 2, 10)$		1.3×10^{2}	2.0×10^{2}	3.8×10^{2}	E
β_E	maximum enzyme concentration	enzymes cell^{-1}	1125	f		fixed value		E
B_0	initial cell concentration	cells L^{-1}	$T(21, 0.5, 10)$	e	3.0×10^{9}	3.3×10^{9}	3.5×10^{9}	M
					3.0×10^{9}	3.3×10^{9}	3.5×10^{9}	E
b_{O_2}	background value of O_2	M	$N(-18, 3)$		8.1×10^{-8}	9.5×10^{-8}	1.1×10^{-7}	M
					8.2×10^{-8}	9.5×10^{-8}	1.1×10^{-7}	E
$b_{NO_2^-}$	background value of NO_2^-	M	$N(-18, 3)$		3.6×10^{-10}	1.0×10^{-8}	1.3×10^{-7}	M
					3.0×10^{-10}	1.2×10^{-8}	1.2×10^{-7}	E
σ_B	constant error of Box-Cox transformed cell density data		Half-Normal(0.1)		2.2×10^{-1}	2.6×10^{-1}	3.1×10^{-1}	M
					2.2×10^{-1}	2.6×10^{-1}	3.2×10^{-1}	E

Continued on the next page.

Table S2: (continued) Simulation parameters, their prior distributions or fixed values, and their posterior medians and percentiles.

Symbol	Description	Units	Prior[a]/Value	Reference	Posterior Percentiles 10 %	Median	90 %	Model[b]
σ_{O_2}	constant error of Box-Cox transformed O_2 gas data		$H(0, 0.1)$		2.5×10^{-2}	2.9×10^{-2}	3.3×10^{-2}	M
					2.6×10^{-2}	2.9×10^{-2}	3.4×10^{-2}	E
$\sigma_{NO_2^-}$	constant error of Box-Cox transformed NO_2^- data		$H(0.025, 0.1)$		2.5×10^{-2}	2.5×10^{-2}	2.6×10^{-2}	M
					2.5×10^{-2}	2.5×10^{-2}	2.6×10^{-2}	E
σ_{N_2}	constant error of Box-Cox transformed N_2 gas data		$H(0.1, 0.1)$		1.0×10^{-1}	1.0×10^{-1}	1.0×10^{-1}	M
					1.0×10^{-1}	1.0×10^{-1}	1.0×10^{-1}	E
σ_{nar}	constant error of Box-Cox transformed *nar* mRNA data		$H(0, 0.1)$		8.7×10^{-3}	4.0×10^{-2}	9.9×10^{-2}	E
σ_{nir}	constant error of Box-Cox transformed *nir* mRNA data		$H(0, 0.1)$		3.1×10^{-2}	4.2×10^{-2}	5.7×10^{-2}	E
λ_B	Box-Cox transformation parameter of the cell densities		5×10^{-2}			fixed value		E, M
$\lambda_{NO_2^-}$	Box-Cox transformation parameter of the NO_2^- data		4×10^{-1}			fixed value		E, M
λ_{N_2}	Box-Cox transformation parameter of the N_2 data		1.8×10^{-1}			fixed value		E, M
λ_{O_2}	Box-Cox transformation parameter of the O_2 data		1.8×10^{-1}			fixed value		E, M
λ_{nar}	Box-Cox transformation parameter of the *nar* mRNA data		1×10^{-1}			fixed value		E
λ_{nir}	Box-Cox transformation parameter of the *nir* mRNA data		4×10^{-1}			fixed value		E

[a] Prior distributions are defined for the log of all parameters except for σ_i . $N(\mu, \sigma)$ is a normal distribution, $H(\mu, \sigma)$ is a half-normal distribution and $T(\mu, \sigma, \nu)$ a Student's *t*-distribution with location μ, scale σ and degrees of freedom ν. [b] E means *enzyme-based model* and M means *Monod-type model*
[c] Parameters for transcriptional regulation are not well known. We chose very broad distributions spanning several orders of magnitude. [d] Reparametrization of v^i_{max}, see section 4 for details. [e] Based on experimental data. [f] Maier et al. (2011) [g] Roberts et al. (2011) [h] Qu et al. (2015) [i] Suenaga et al. (2018) [j] Hassan et al. (2016) [k] Lee et al. (2006)

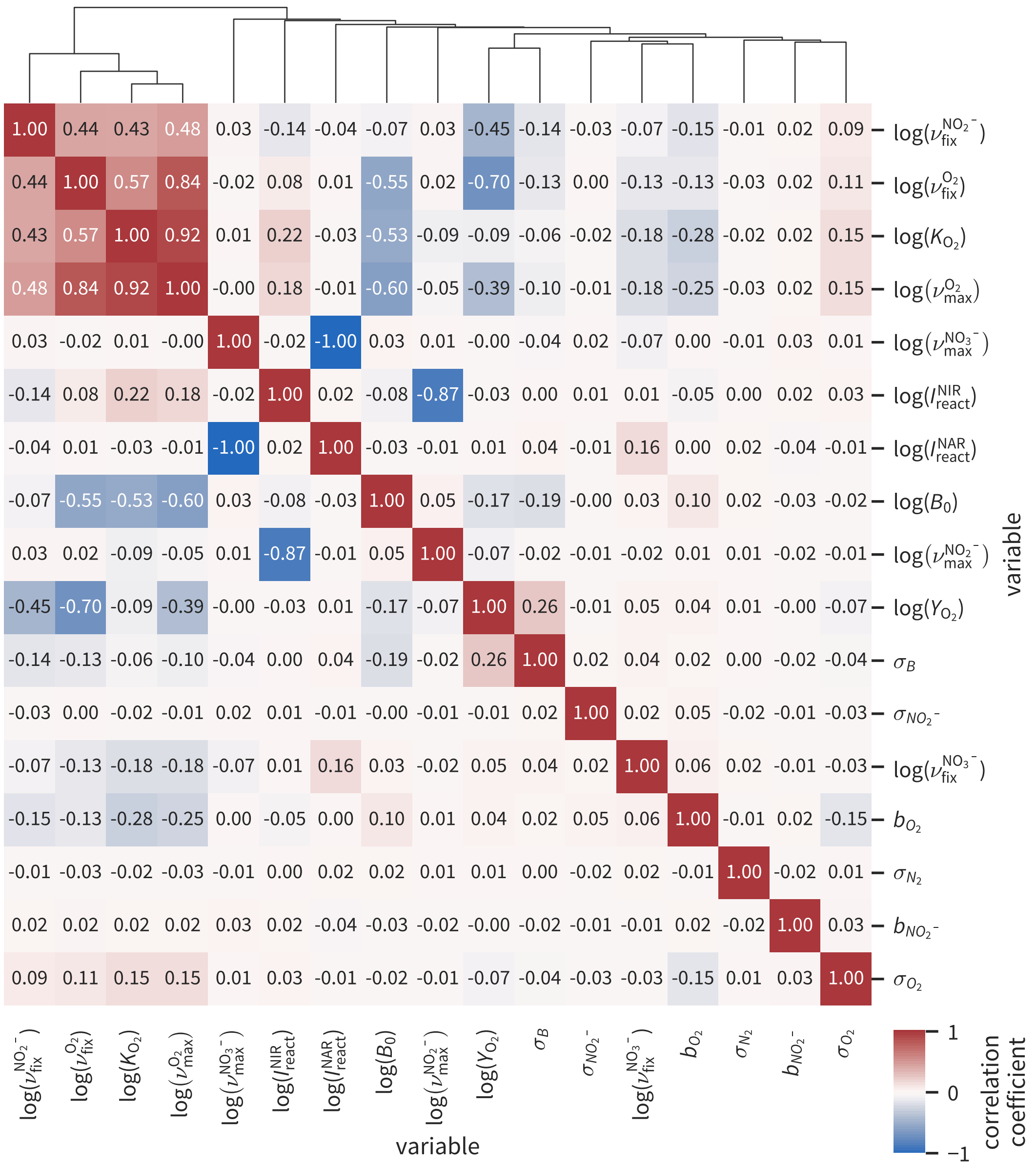

Figure S2: Hierarchically-clustered heatmap showing the correlation coefficients of the estimated parameters in the posterior distribution of the Monod-type model.

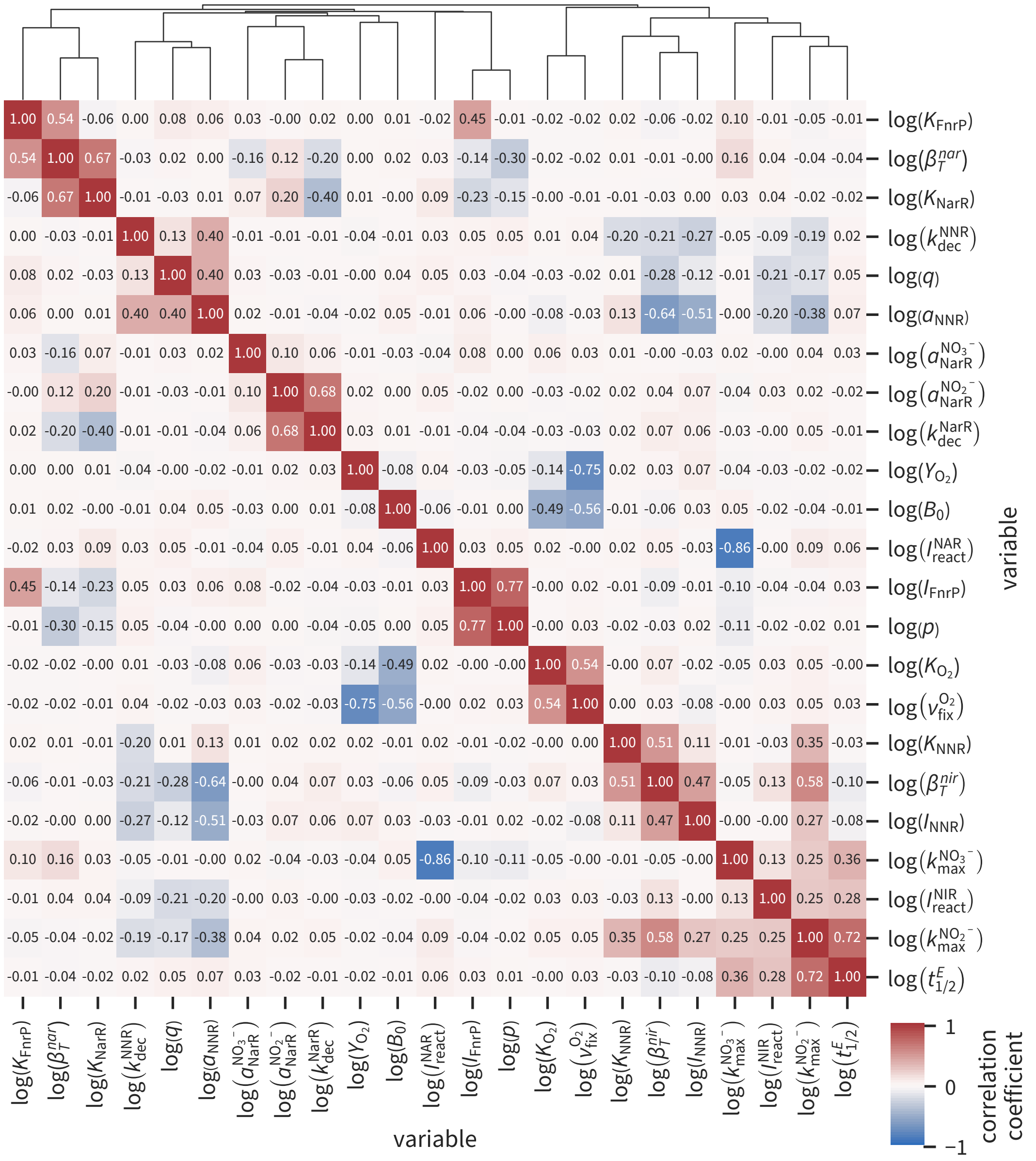

Figure S3: Hierarchically-clustered heatmap showing the correlation coefficients of the estimated parameters in the posterior distribution of the enzyme-based model.

3 Transcription Factor Concentrations

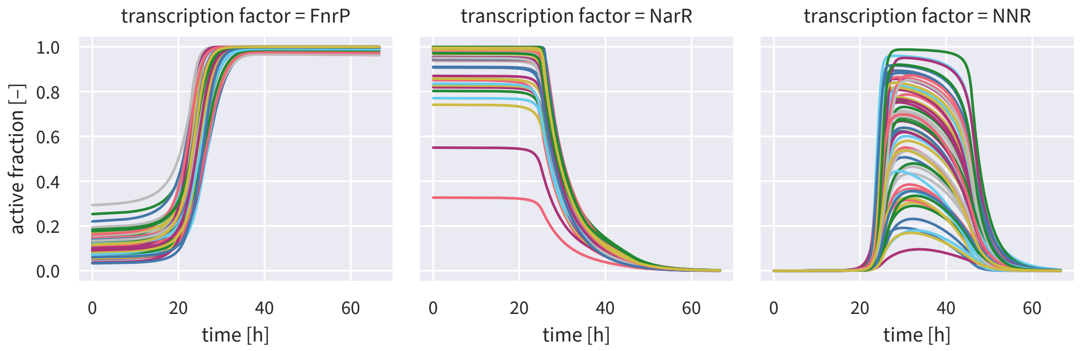

Figure S4: Simulated fraction of active transcription factors over time. Several draws from the posterior are indicated by color.

4 Reparametrization of Monod Parameters

The Monod parameters of aerobic respiration, K_{O_2} and $v_{max}^{O_2}$, showed a strong correlation in the posterior. Heavy parameter correlations can hinder effective sampling from the posterior. We therefore decided to apply a reparametrization that describes the same rate law in terms of different parameters. $v_{max}^{O_2}$ can be interpreted as the cell-specific reaction rate at the limit of the oxygen concentration going to infinity. Instead of using the rate at the limit, we use the rate at a fixed finite oxygen concentration, $C_{O_2}^{fix}$:

$$v_{fix}^{O_2} = v_{max}^{O_2} \frac{C_{O_2}^{fix}}{C_{O_2}^{fix} + K_{O_2}} . \tag{1}$$

We can solve equation (1) for the maximum cell specific rate $v_{max}^{O_2}$, expressing it in terms of K_{O_2}, $C_{O_2}^{fix}$ and $v_{fix}^{O_2}$. We chose $C_{O_2}^{fix}$ manually such that the correlation between $v_{fix}^{O_2}$ and K_{O_2} is low.

We apply a similar reparametrization for the maximum cell-specific denitrification rates and oxygen inhibition constants in the Monod-type model. The maximum cell-specific rate v_{max}^{i} of nitrogen substrate i is the rate at the limit of the substrate concentration reaching infinity and an oxygen concentration of zero. Instead, we use the cell-specific rate at a fixed, non-zero oxygen concentration $C_{O_2}^{fix,i}$ and without substrate limitation (i.e. $C_i \to \infty$) as parameter:

$$v_{fix}^{i} = v_{max}^{i} \frac{I_{reac}^{i}}{I_{reac}^{i} + C_{O_2}^{fix,i}} . \tag{2}$$

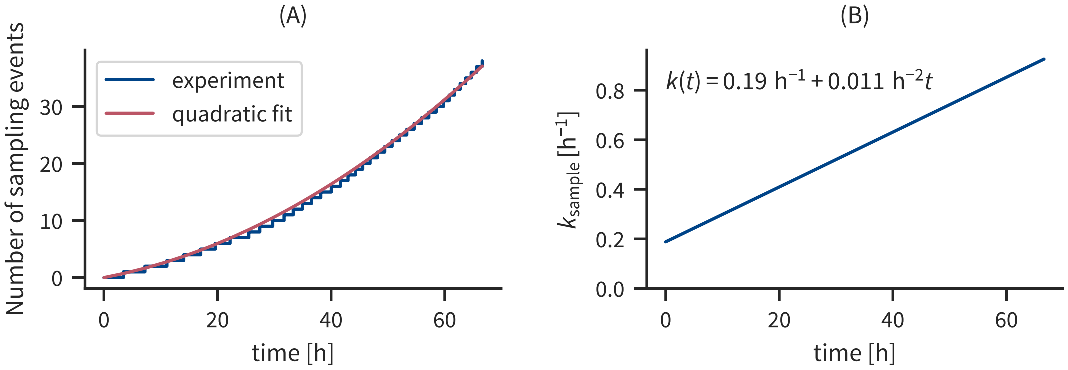

Figure S5: **(A)** Quadratic fit to the number of sampling events in the experiment. **(B)** The derivative of the function gives the time dependent coefficient for sampling related rates k_{sample}.

5 Mass Transfer to the Gas Phase and Gas Sampling

N_2 and O_2 are gases that partition into the headspace. The flasks used in the experiment had a headspace. Therefore, the model needs to account for the partitioning of molecular nitrogen and oxygen between the water and gas phases. Henry's law describes the relation between aqueous and gas phase concentrations of compound i (C^i_{w} and C^i_{g}) at equilibrium:

$$C^i_{\mathrm{g}} = H_i C^i_{\mathrm{w}}\,, \tag{3}$$

with the Henry's-law coefficient $H_i[\mathrm{mol\,L_{gas}^{-1}\,mol^{-1}\,L_{liq}}]$. Kinetic mass transfer of compound i between the two phases is described by a linear driving force model with rate coefficient $k^i_{\mathrm{tr}}\,[\mathrm{s}^{-1}]$. The transfer rate r^i_{tr} $[\mathrm{mol\,L_{liq}^{-1}\,s^{-1}}]$, expressed as the mass-transfer related concentration change in the liquid phase, is then given by

$$r^i_{\mathrm{tr}} = k^i_{\mathrm{tr}}\left(\frac{C^i_{\mathrm{g}}}{H_i} - C^i_{\mathrm{w}}\right). \tag{4}$$

In the experiment, the head space was sampled in regular intervals. Helium was subsequently injected to compensate the pressure decrease resulting from the removal of gas. This led to a dilution of the gas-phase concentrations, which we describe by a first order rate law:

$$r^i_{\mathrm{sample}} = f_{\mathrm{dil}} k_{\mathrm{sample}}(t)\, C^i_{\mathrm{g}}\,, \tag{5}$$

in which f_{dil} is the fraction of gas exchanged at each sampling event. The function $k_{\mathrm{sample}}(t)[\mathrm{s}^{-1}]$ is a scaled rate constant. It would most appropriately be described by several pulses, each integrating to one. To avoid discontinuities we replaced it by a linear function with approximately the same integral (figure S5).

Following Qu et al. (2015) we also account for small leakage rates r^i_{leak} of N_2 and O_2 into the system, with a constant diffusion rate r_{diff} $[\mathrm{mol\,L_{gas}^{-1}\,s^{-1}}]$ and a time-dependent rate that

Table S3: Parameters related to mass transfer and gas sampling with their values and units.

Symbol	Description	Value	Units	Reference
k_{tr}	mass transfer coefficient	0.08	s^{-1}	a
H_{O_2}	Henry's coefficient of O_2 at 20 °C	31.2	$M\,M^{-1}$	b
H_{N_2}	Henry's coefficient of N_2 at 20 °C	59.5	$M\,M^{-1}$	b
V_g	volume of the gas phase	0.07	L	c
V_w	volume of the liquid phase	0.05	L	c
f_{dil}	volume fraction replaced per sampling	0.013	–	a
$r_{diff}^{O_2}$	diffusion rate of O_2 into the system	2.7×10^{-12}	$M\,s^{-1}$	a
$r_{diff}^{N_2}$	diffusion rate of N_2 into the system	3.5×10^{-12}	$M\,s^{-1}$	a
$C_{leak}^{O_2}$	O_2 concentration increase per sampling	4.2×10^{-8}	M	a
$C_{leak}^{N_2}$	N_2 concentration increase per sampling	3.5×10^{-7}	M	a

[a] Linda Bergaust (personal communication, 2018-11-05) [b] Sander (2015) [c] Qu et al. (2015)

accounts for higher leakage during sampling:

$$r_{leak}^{i} = r_{diff}^{i} + k_{sample}(t) C_{leak}^{i}\,, \tag{6}$$

in which C_{leak}^{i} [mol L_{gas}^{-1}] is the concentration increase of compound i during a single sampling event. The rate of change of gas-phase concentrations is then given by

$$\frac{dC_g^i}{dt} = r_{leak}^{i} - r_{sample}^{i} - \frac{V_w}{V_g} r_{tr}^{i}\,, \tag{7}$$

with V_g and V_w being the volume of the headspace and the liquid phase, respectively.

6 Simplified Model of Transcriptional Regulation

6.1 Model Equations

In addition to the model that represents the transcription factors FnrP, NarR and NNR explicitly we set up a simplified model variant where transcriptional regulation directly depends on nitrogen oxide and oxygen concentrations. As we will show, however, this formulation can also be interpreted mechanistically in terms of regulation by transcription factors if some simplifying assumptions are made. The necessary assumptions partly differ from those made in the model that explicitly simulates transcription factor concentrations and are discussed in section 6.3.

We assume that the transcription rate scales with the fraction of operator sites where an activating, but no repressing transcription factor is bound. Transcription factors can only bind

to the operator when they are activated by a signaling molecule: N-substrates in the case of activators and oxygen for inhibitors. In our formulation of transcription kinetics, we assume that concentrations of activated transcription factors are proportional to the concentrations of the respective signaling compounds. The transcription rate for gene *i* is

$$r^i_{\mathrm{transcription}} = \alpha f^i_{\mathrm{act}} f^i_{\mathrm{inh}} B, \tag{8}$$

in which α [transcripts cell^{-1} s^{-1}] is the maximum transcription rate, and B is the cell density [cells L^{-1}].

The dimensionless factors f_{act} and f_{inh} (ranging between 0 and 1) regulate the transcription rate as influenced by external factors. The subscript "act" denotes an activator compound. For the case of *nirG* transcription, NO acts as the single activator compound (Bergaust et al., 2012; Spiro, 2012). Analogously to our description of NNR activation, however, we used nitrite as a proxy activator compound because we describe denitrification as a two-step reaction and do not explicitly simulate NO. Thus, the regulation factor for *nir* transcription is

$$f^{nir}_{\mathrm{act}} = \frac{C_{\mathrm{NO_2^-}}}{A^{\mathrm{NO_2^-}}_{nir} + C_{\mathrm{NO_2^-}}}, \tag{9}$$

in which $C_{\mathrm{NO_2^-}}$ is the concentration of nitrite [M] and $A^{\mathrm{NO_2^-}}_{nir}$ is the half-velocity constant of transcription, that is, the concentration of the activator compound nitrite at which the transcription reaches half of its maximum rate (in absence of repressors). In contrast, transcription of *narG* in *P. denitrificans* can be activated by either nitrate or nitrite (Wood et al., 2001):

$$f^{nar}_{\mathrm{act}} = \frac{\frac{C_{\mathrm{NO_3^-}}}{A^{\mathrm{NO_3^-}}_{nar}} + \frac{C_{\mathrm{NO_2^-}}}{A^{\mathrm{NO_2^-}}_{nar}}}{1 + \frac{C_{\mathrm{NO_3^-}}}{A^{\mathrm{NO_3^-}}_{nar}} + \frac{C_{\mathrm{NO_2^-}}}{A^{\mathrm{NO_2^-}}_{nar}}}, \tag{10}$$

in which $C_{\mathrm{NO_3^-}}$ is the concentration of nitrate [M] whereas $A^{\mathrm{NO_3^-}}_{nar}$ and $A^{\mathrm{NO_2^-}}_{nar}$ are the half-velocity constants of the activator compounds nitrate and nitrite, respectively.

Transcription of the *nirS* and *narG* genes is inhibited by the presence of oxygen, described by the oxygen transcriptional inhibition factor, f^i_{inh}:

$$f^i_{\mathrm{inh}} = \frac{I^i_{\mathrm{trs}}}{C_{\mathrm{O_2}} + I^i_{\mathrm{trs}}}, \tag{11}$$

in which $C_{\mathrm{O_2}}$ is the concentration of oxygen [M] and I^i_{trs} is the transcript inhibition constant of oxygen for gene *i*. In contrast to the model that represents transcription factors explicitly, we described transcript concentrations by the transient equation instead of a quasi-steady state.

6.2 Simulation Results

Figure S6 shows the simulation results obtained with the simplified model formulation. Table S5 lists the prior distributions and statistics of posterior distributions for all estimated parameters used in the simplified model simulation. Fixed parameters have the same values as in table S2 and are therefore omitted.

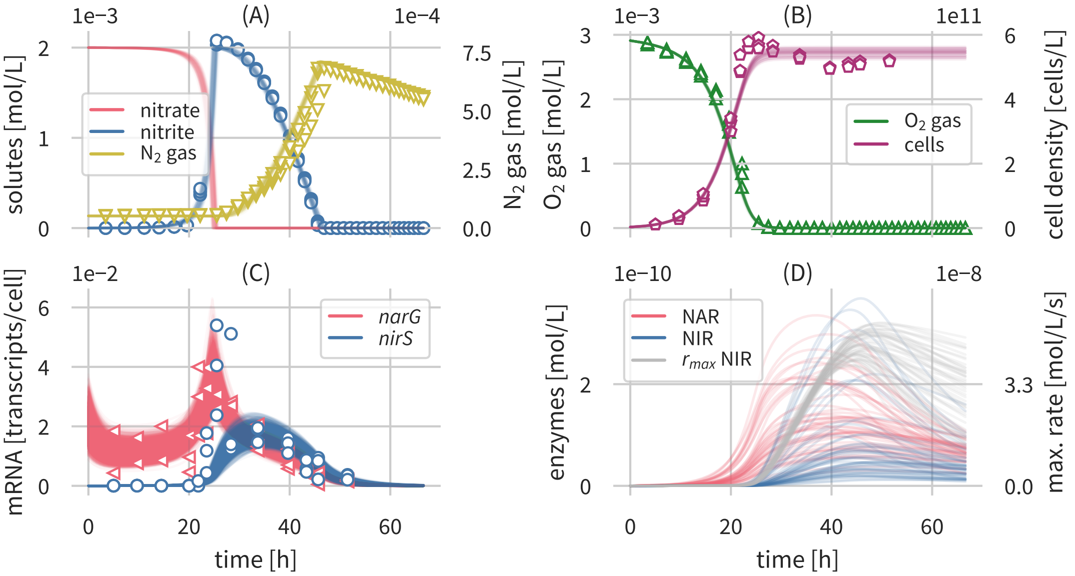

Figure S6: Measurement data, 50 draws from the posterior of the enzyme-based model with a simplified description of transcirptional regulation as presented in section 6.1: **(A)** Nitrogen compounds, **(B)** oxygen and cell densities, **(C)** transcripts, **(D)** enzymes and maximum rates.

Table S5: Simulation parameters of the enzyme-based model with a simplified formulation for transcription, their prior distributions, and their posterior medians and percentiles. Fixed parameters have te same values as in table S2 and are omitted.

Symbol	Description	Units	Prior[a]/Value	Reference	Posterior Percentiles 10 %	Posterior Percentiles Median	Posterior Percentiles 90 %
$t^{T}_{1/2}$	transcript half life	min	$N(1.8, 0.4)$	c, d	1.3×10^{2}	1.6×10^{2}	1.9×10^{2}
$A^{NO_3^-}_{nar}$	NO_3^- concentration inducing transcription of *nar*	M	$N(-12, 2)$	b	4.3×10^{-7}	5.9×10^{-6}	7.1×10^{-5}
$A^{NO_2^-}_{nar}$	NO_2^- concentration inducing transcription of *nar*	M	$N(-8, 2)$	b	4.8×10^{-3}	8.5×10^{-3}	1.6×10^{-2}
$A^{NO_2^-}_{nir}$	NO_2^- concentration inducing transcription of *nir*	M	$N(-11, 3)$	b	2.6×10^{-3}	7.8×10^{-3}	2.0×10^{-2}
I^{nar}_{trs}	O_2 inhibition parameter for *nar*-transcription	M	$T(-11, 2, 10)$	b	1.4×10^{-5}	4.2×10^{-5}	8.6×10^{-5}
I^{nir}_{trs}	O_2 inhibition parameter for *nir*-transcription	M	$T(-11, 2, 10)$	b	1.9×10^{-5}	4.8×10^{-5}	3.5×10^{-4}
$t^{E}_{1/2}$	enzyme half life	h	$N(3, 0.8)$	g, h	1.1×10^{1}	2.2×10^{1}	4.9×10^{1}
β^{nar}_{T}	maximum *narG* concentration	transcripts cell^{-1}	$T(-2, 0.7, 10)$	g, i	4.8×10^{-2}	6.7×10^{-2}	1.1×10^{-1}
β^{nir}_{T}	maximum *nirS* concentration	transcripts cell^{-1}	$T(-2, 0.7, 10)$	g, i	4.4×10^{-2}	1.0×10^{-1}	2.2×10^{-1}
I^{NAR}_{reac}	O_2 inhibition parameter for reaction of NO_3^-	M	$N(-15, 3)$		1.3×10^{-7}	8.7×10^{-7}	3.6×10^{-6}
I^{NIR}_{reac}	O_2 inhibition parameter for reaction of NO_2^-	M	$N(-15, 3)$		1.8×10^{-7}	1.5×10^{-6}	3.4×10^{-5}
K_{O_2}	O_2 half-saturation constant	M	$T(-13, 2, 10)$	j	2.9×10^{-5}	3.2×10^{-5}	3.5×10^{-5}
Y_{O_2}	O_2 growth yield per amount e^--donor	cells mol^{-1}	$T(32.2, 0.5, 10)$	k	4.8×10^{14}	4.9×10^{14}	5.0×10^{14}
$v^{O_2}_{fix}$	O_2 reaction rate at fixed O_2 concentration	mol cell^{-1} s^{-1}	$T(-44.2, 1.6, 10)$	e	4.1×10^{-19}	4.2×10^{-19}	4.3×10^{-19}
$k^{NO_3^-}_{max}$	NAR turnover number	s^{-1}	$T(7, 2, 10)$		7.8×10^{3}	3.8×10^{4}	2.7×10^{5}
$k^{NO_2^-}_{max}$	NIR turnover number	s^{-1}	$T(7, 2, 10)$		2.9×10^{2}	7.2×10^{2}	1.9×10^{3}
B_0	initial cell concentration	cells L^{-1}	$T(21, 0.5, 10)$	f	3.0×10^{9}	3.3×10^{9}	3.5×10^{9}
b_{O_2}	background value of O_2	M	$N(-18, 3)$		8.1×10^{-8}	9.5×10^{-8}	1.1×10^{-7}

Continued on the next page.

Table S5: (continued) Simulation parameters of the enzyme-based model with a simplified formulation for transcription, their prior distributions, and their posterior medians and percentiles. Fixed parameters have te same values as in table S2 and are omitted.

Symbol	Description	Units	Prior[a]/Value	Reference	Posterior Percentiles 10 %	Median	90 %
$b_{NO_2^-}$	background value of NO_2^-	M	$N(-18, 3)$		3.4×10^{-10}	1.1×10^{-8}	1.3×10^{-7}
σ_B	constant error of Box-Cox transformed cell density data		Half-Normal(0.1)		2.1×10^{-1}	2.6×10^{-1}	3.1×10^{-1}
σ_{O_2}	constant error of Box-Cox transformed O_2 gas data		$H(0, 0.1)$		2.5×10^{-2}	2.9×10^{-2}	3.3×10^{-2}
$\sigma_{NO_2^-}$	constant error of Box-Cox transformed NO_2^- data		$H(0.025, 0.1)$		2.5×10^{-2}	2.5×10^{-2}	2.6×10^{-2}
σ_{N_2}	constant error of Box-Cox transformed N_2 gas data		$H(0.1, 0.1)$		1.0×10^{-1}	1.0×10^{-1}	1.0×10^{-1}
σ_{nar}	constant error of Box-Cox transformed *nar* mRNA data		$H(0, 0.1)$		1.1×10^{-1}	1.6×10^{-1}	2.2×10^{-1}
σ_{nir}	constant error of Box-Cox transformed *nir* mRNA data		$H(0, 0.1)$		2.9×10^{-2}	4.1×10^{-2}	5.7×10^{-2}

[a] Prior distributions are defined for the log of all parameters but σ_i . $N(\mu, \sigma)$ is a normal distribution, $H(\mu, \sigma)$ is a half-normal distribution and $T(\mu, \sigma, \nu)$ a Student's *t*-distribution with location μ, scale σ and degrees of freedom ν. [b] Parameters for transcriptional regulation are not well known. We chose very broad distributions spanning several orders of magnitude. [c] Bernstein et al. (2002) [d] Härtig and Zumft (1999) [e] Reparametrization of v^i_{max}, see section 4 for details. [f] Based on experimental data. [g] Maier et al. (2011) [h] Roberts et al. (2011) [i] Qu et al. (2015) [j] Suenaga et al. (2018) [k] Hassan et al. (2016)

6.3 Deriving the Transcriptional Regulation From Mechanistic Principles

The formulation of transcriptional regulation presented in section 6.1, even though simplified compared to the actual underlying mechanisms, can be interpreted in terms of mechanistic principles. The derivations follow the analysis of Ingalls (2013, chapter 7.1.2).

6.3.1 One Activator and One Repressor

We consider a gene that is regulated by two transcription factors, an activator A and a repressor R. They can bind at two distinct operator sites of the regulated gene's promoter region. Transcription will only occur if the activator is bound but the repressor is not bound to the operator O. We can describe the binding of the transcription factors at the operator by the following reactions:

$$\mathrm{O} + \mathrm{A} \underset{d_1}{\overset{a_1}{\rightleftharpoons}} \mathrm{OA}\,, \tag{I}$$

$$\mathrm{O} + \mathrm{R} \underset{d_2}{\overset{a_2}{\rightleftharpoons}} \mathrm{OR}\,, \tag{II}$$

$$\mathrm{OR} + \mathrm{A} \underset{d_1}{\overset{a_1}{\rightleftharpoons}} \mathrm{OAR}\,, \tag{III}$$

$$\mathrm{OA} + \mathrm{R} \underset{d_2}{\overset{a_2}{\rightleftharpoons}} \mathrm{OAR}\,, \tag{IV}$$

in which a_1 and a_2 $[\mathrm{M}^{-1}\,\mathrm{s}^{-1}]$ are reaction constants for the forward reaction and d_1, d_2 $[\mathrm{s}^{-1}]$ are the backwards reaction constants. Note that the binding of the two transcriptions factors is assumed to be independent. That is, the binding kinetics of R to O and to OA are the same. Likewise A binds to O and OR with the same rate constants.

The transcription rate can be considered to be proportional to the fraction of operator sites where only the activator is bound, f_{OA}:

$$r_{\text{transcription}} = \alpha \cdot f_{\mathrm{OA}}\,, \tag{12}$$

$$f_{\mathrm{OA}} = \frac{C_{\mathrm{OA}}}{C_{\mathrm{O}} + C_{\mathrm{OA}} + C_{\mathrm{OR}} + C_{\mathrm{OAR}}}\,. \tag{13}$$

Assuming that the binding reactions are at steady state and setting

$$K_A = \frac{d_1}{a_1}, \qquad K_R = \frac{d_2}{a_2}\,, \tag{14}$$

we can write:

$$f_{\mathrm{OA}} = \frac{C_{\mathrm{A}}}{K_A + C_{\mathrm{A}} + \frac{C_{\mathrm{R}}K_A}{K_R} + \frac{C_{\mathrm{A}}C_{\mathrm{R}}}{K_R}} = \frac{C_{\mathrm{A}}}{C_{\mathrm{A}} + K_A}\frac{K_R}{K_R + C_{\mathrm{R}}}\,. \tag{15}$$

The transcription factor concentrations react to changes in environmental conditions. In a simplified model, this interaction could be described as the reaction of an inactive form of the

transcription factor with an activating molecule. In the case of the denitrification genes, we assume that a nitrogen substrate N reacts with the activator and oxygen with the repressor:

$$\mathrm{N} + \mathrm{A}^* \xrightleftharpoons{K_1} \mathrm{A} \tag{V}$$

$$\mathrm{O_2} + \mathrm{R}^* \xrightleftharpoons{K_2} \mathrm{R}\,. \tag{VI}$$

Here, the inactive form of the transcription factors are indicated by an asterisk. We now assume: (i) The activation and deactivation reactions follow first order kinetics with respect to N or O_2 and A or R, respectively. (ii) Activation and deactivation of transcription factors are fast compared to gene transcription, i.e. A and R are in quasi-steady state. The concentrations of active transcription factors, C_A and C_R [M], then are proportional to the nitrogen substrate and oxygen concentrations:

$$C_\mathrm{A} = m_\mathrm{A} \cdot C_\mathrm{N}\,, \tag{16}$$

$$C_\mathrm{R} = m_\mathrm{R} \cdot C_{\mathrm{O_2}}\,, \tag{17}$$

with the proportionality factors m_A and m_R [mol mol^{-1}]. Substituting these expressions into equation (15) we obtain

$$f_\mathrm{OA} = \frac{C_\mathrm{N}}{C_\mathrm{N} + \frac{K_A}{m_\mathrm{A}}} \cdot \frac{\frac{K_R}{m_\mathrm{R}}}{\frac{K_R}{m_\mathrm{R}} + C_{\mathrm{O_2}}} = \frac{C_\mathrm{N}}{C_\mathrm{N} + A_\mathrm{N}} \cdot \frac{I_\mathrm{trs}^{\mathrm{O_2}}}{I_\mathrm{trs}^{\mathrm{O_2}} + C_{\mathrm{O_2}}}\,, \tag{18}$$

with the transcription half-saturation constant A_N for substrate N and the oxygen inhibition constant for transcription $I_\mathrm{trs}^{\mathrm{O_2}}$. Combining equations (12) and (18) leads to our formulation of the *nir* transcription rate given in equation (9).

6.3.2 Two Activators and One Repressor

The transcription of some genes might be activated by several different compounds. We can describe this with two different mechanisms which eventually lead to the same rate law for the transcription rate: (i) There is a single activating transcription factor A binding to the operator site. The concentration of the activator is determined by the concentrations of both activating compounds. (ii) There are two different activating transcription factors A_1 and A_2 that can both bind to the same operating site, i.e. either A_1 *or* A_2 can be bound. The concentration of each transcription factor is determined by the concentration of one of the activating compounds. In the latter case the transcription rate is proportional to the fraction of operator sites in the states OA_1 and OA_2. This fraction is given by

$$f_{\mathrm{OA_1,OA_2}} = \frac{C_{\mathrm{OA_1}} + C_{\mathrm{OA_2}}}{C_\mathrm{O} + C_{\mathrm{OA_1}} + C_{\mathrm{OA_2}} + C_{\mathrm{OA_1R}} + C_{\mathrm{OA_2R}} + C_\mathrm{OR}}\,. \tag{19}$$

Assuming equilibrium conditions like in equation (14) with K_{A_1}, K_{A_2} and K_R being the equilibrium constants for the binding of A_1, A_2, and R we obtain

$$f_{OA_1,OA_2} = \frac{K_R}{K_R + C_R} \cdot \frac{\frac{C_{A_1}}{K_{A_1}} + \frac{C_{A_2}}{K_{A_2}}}{1 + \frac{C_{A_1}}{K_{A_1}} + \frac{C_{A_2}}{K_{A_2}}} . \tag{20}$$

Assuming that the transcription factor concentrations follow

$$C_{A_1} = m_{A_1} \cdot C_{N_1} , \tag{21}$$
$$C_{A_2} = m_{A_2} \cdot C_{N_2} , \tag{22}$$
$$C_R = m_R \cdot C_{O_2} , \tag{23}$$

where N_1 and N_2 denote the two inducing nitrogen substrates, and setting

$$A_{N_1} = \frac{K_{A_1}}{m_{A_1}} , \qquad A_{N_2} = \frac{K_{A_2}}{m_{A_2}} , \qquad I_{trs}^{O_2} = \frac{K_R}{m_R} , \tag{24}$$

we can write:

$$f_{OA_1,OA_2} = \frac{I_{trs}^{O_2}}{I_{trs}^{O_2} + C_{O_2}} \cdot \frac{\frac{C_{N_1}}{A_{N_1}} + \frac{C_{N_2}}{A_{N_2}}}{1 + \frac{C_{N_1}}{A_{N_1}} + \frac{C_{N_2}}{A_{N_2}}} . \tag{25}$$

This is the formulation that we used for the transcription of *nar*, which is triggered by nitrate or nitrite given in equation (10).

6.3.3 Discussion of the Underlying Assumptions

We argue that a linear relation between signalling compounds and transcription factor concentrations is a reasonable simplifying assumption. It enables the description of the regulation mechanism with a single parameter per transcription factor. However, when interpreting results based on this assumption one should keep its implications in mind:

1. First-order kinetics for the activation reaction of the transcription factor as given in equations (21) to (23) require that the concentration of inactive transcription factors is not limiting for the binding reaction, that is, C_N and C_{O_2} must be small compared to the equilibrium constant of the activation reaction K. Otherwise, assuming a constant total amount of transcription factors (active and inactive) C_A^{tot}, the relationship would be of the form

$$C_A = C_A^{tot} \frac{C_N}{C_N + K} \tag{26}$$

   instead of a linear relationship.

2. Assuming a quasi-steady state of the active transcription factors is only valid if the activation or deactivation are much faster than substrate dynamics and transcription itself. Crack et al. (2016) showed that the deactivation of the oxygen-sensitive transcription factor FnrP by exposure to oxygen takes several minutes in *P. denitrificans* (mean lifetime of 3.5 min), which is similar to literature mRNA half-lives. Our simulation results reproduce the continued presence of transcripts after all substrate is used up. However, it does not resolve the dynamics of transcription factors and transcripts separately due to the quasi-steady state assumption. As a consequence, the mRNA half-life acts as a lumped parameter that accounts for both effects (non-immediate inactivation of transcription factors and decay of transcripts) and is much longer than expected from the literature.

References

Bergaust, L., van Spanning, R. J. M., Frostegård, Å., & Bakken, L. R. (2012). Expression of nitrous oxide reductase in *Paracoccus denitrificans* is regulated by oxygen and nitric oxide through FnrP and NNR. *Microbiology (Reading, England)*, *158*, 826–834. https://doi.org/10.1099/mic.0.054148-0

Bernstein, J. A., Khodursky, A. B., Lin, P.-H., Lin-Chao, S., & Cohen, S. N. (2002). Global analysis of mRNA decay and abundance in *Escherichia coli* at single-gene resolution using two-color fluorescent DNA microarrays. *Proceedings of the National Academy of Sciences of the United States of America*, *99*(15), 9697–9702. https://doi.org/10.1073/pnas.112318199

Crack, J. C., Hutchings, M. I., Thomson, A. J., & Le Brun, N. E. (2016). Biochemical properties of *Paracoccus denitrificans* FnrP: Reactions with molecular oxygen and nitric oxide. *JBIC Journal of Biological Inorganic Chemistry*, *21*(1), 71–82. https://doi.org/10.1007/s00775-015-1326-7

Härtig, E., & Zumft, W. G. (1999). Kinetics of *nirS* expression (cytochrome cd_1 nitrite reductase) in *Pseudomonas stutzeri* during the transition from aerobic respiration to denitrification: Evidence for a denitrification-specific nitrate- and nitrite-responsive regulatory system. *Journal of Bacteriology*, *181*(1), 161–166. https://doi.org/10.1128/JB.181.1.161-166.1999

Hassan, J., Qu, Z., Bergaust, L. L., & Bakken, L. R. (2016). Transient accumulation of $NO_2{}^-$ and N_2O during denitrification explained by assuming cell diversification by stochastic transcription of denitrification genes. *PLoS Computational Biology*, *12*(1). https://doi.org/10.1371/journal.pcbi.1004621

Ingalls, B. P. (2013). *Mathematical modeling in systems biology: An introduction*. MIT Press OCLC: 884833409.

Lee, Y.-Y., Shearer, N., & Spiro, S. (2006). Transcription factor NNR from *Paracoccus denitrificans* is a sensor of both nitric oxide and oxygen: Isolation of *nnr** alleles encoding effector-independent proteins and evidence for a haem-based sensing mechanism. *Microbiology*, *152*(5), 1461–1470. https://doi.org/10.1099/mic.0.28796-0

Maier, T., Schmidt, A., Güell, M., Kühner, S., Gavin, A.-C., Aebersold, R., & Serrano, L. (2011). Quantification of mRNA and protein and integration with protein turnover in a bacterium. *Molecular Systems Biology*, *7*, 511. https://doi.org/10.1038/msb.2011.38

Qu, Z., Bakken, L. R., Molstad, L., Frostegård, Å., & Bergaust, L. L. (2015). Transcriptional and metabolic regulation of denitrification in *Paracoccus denitrificans* allows low but significant activity of nitrous oxide reductase under oxic conditions. *Environmental Microbiology*, *18*(9), 2951–2963. https://doi.org/10.1111/1462-2920.13128

Roberts, E., Magis, A., Ortiz, J. O., Baumeister, W., & Luthey-Schulten, Z. (2011). Noise contributions in an inducible genetic switch: A whole-cell simulation study. *PLoS Computational Biology*, *7*(3). https://doi.org/10.1371/journal.pcbi.1002010

Sander, R. (2015). Compilation of Henry's law constants (version 4.0) for water as solvent. *Atmospheric Chemistry and Physics*, *15*(8), 4399–4981. https://doi.org/10.5194/acp-15-4399-2015

Spiro, S. (2012). Nitrous oxide production and consumption: Regulation of gene expression by gas-sensitive transcription factors. *Philosophical Transactions of the Royal Society B: Biological Sciences*, *367*(1593), 1213–1225. https://doi.org/10.1098/rstb.2011.0309

Suenaga, T., Riya, S., Hosomi, M., & Terada, A. (2018). Biokinetic characterization and activities of N_2O-reducing bacteria in response to various oxygen levels. *Frontiers in Microbiology*, *9*. https://doi.org/10.3389/fmicb.2018.00697

Wood, N. J., Alizadeh, T., Bennett, S., Pearce, J., Ferguson, S. J., Richardson, D. J., & Moir, J. W. B. (2001). Maximal expression of membrane-bound nitrate reductase in *Paracoccus* is induced by nitrate via a third FNR-like regulator named NarR. *Journal of Bacteriology*, *183*(12), 3606–3613. https://doi.org/10.1128/JB.183.12.3606-3613.2001
